# RTx-303, an Orally Bioavailable Polθ Polymerase Inhibitor That Potentiates PARP Inhibitors in BRCA Mutant Tumors

**DOI:** 10.1021/acs.jmedchem.5c00551

**Published:** 2025-10-22

**Authors:** Gurushankar Chandramouly, William Fried, John Gordon, Douglas Ralph, Channita Keuk, Sangeeta Kumari, Mercy Ramanjulu, William Auerbacher, Leonid Minakhin, Taylor Tredinnick, Bernadette Tiberi, George Morton, Robert Betsch, Kathy Q. Cai, Umeshkumar M. Vekariya, Mrityunjay Tyagi, Tomasz Skorski, Sergey Karakashev, Neil Johnson, Wayne E. Childers, Xiaojiang S. Chen, Richard T. Pomerantz

**Affiliations:** † Molecular and Computational Biology, USC Dornsife Department of Biological Sciences, 5116 University of Southern California, Los Angeles, California 90089, United States; ‡ Department of Biochemistry and Molecular Biology, Sidney Kimmel Cancer Center, 29445 Thomas Jefferson University, Philadelphia, Pennsylvania 19107, United States; § Recombination Therapeutics, Pennsylvania Biotechnology Center, Doylestown, Pennsylvania 18902, United States; ∥ Nuclear Dynamics and Cancer Program, 6565 Fox Chase Cancer Center, Philadelphia, Pennsylvania 19111, United States; ⊥ Fels Cancer Institute for Personalized Medicine, 110059 Temple University Lewis Katz School of Medicine, Philadelphia, Pennsylvania 19140, United States; # 15493 Temple University, School of Pharmacy, Philadelphia, Pennsylvania 19140, United States

## Abstract

DNA polymerase θ (Polθ) is a polymerase-helicase fusion protein that is synthetically lethal with homologous recombination (HR) factors, such as BRCA1/2, and confers resistance to PARP inhibitors (PARPi) and other genotoxic cancer therapies. Previously developed Polθ polymerase (Polθ-pol) inhibitors (Polθi) exhibited limited pharmacological activity and metabolic stability, warranting the development of a Polθi with improved drug-like properties. Here, we developed RTx-303, a selective allosteric small-molecule Polθ-pol inhibitor that exhibits 5.1 nM IC_50_, 88% oral bioavailability, and a prolonged half-life along with its equipotent metabolite. X-ray crystallography highlights the development of a solvent-exposed side-chain that is essential for the optimal drug-like properties of RTx-303. Notably, RTx-303 exhibits significantly higher cellular potency than previously developed Polθ-pol inhibitors and strongly potentiates PARPi in BRCA1/2 mutant cells and patient-derived xenograft models. The superior potency, robust pharmacological activity, and high tolerability of RTx-303 warrant further development as a Polθ-pol inhibitor drug candidate.

## Graphical abstract

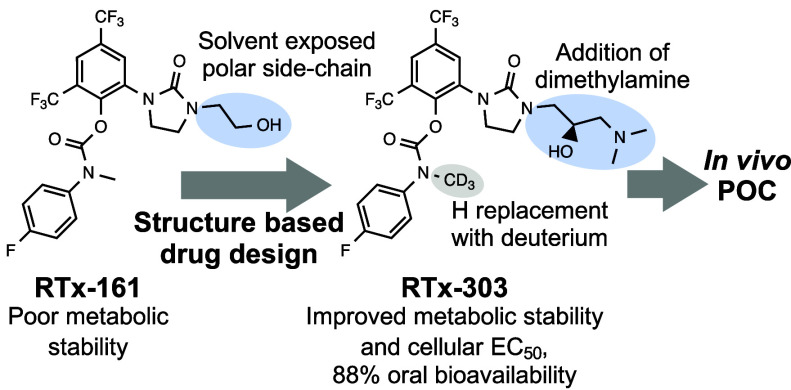

## Introduction

BRCA1 and BRCA2 promote repair of DNA double-strand breaks (DSBs) by facilitating homologous recombination (HR).^1,2^ Yet BRCA1/2 (BRCA) are often mutated in cancers, such as subsets of breast, ovarian, prostate and pancreatic carcinomas.^2–4^ BRCA mutant cancer cells are therefore HR-deficient (HRD), and as a result, highly susceptible to agents that induce DNA damage and/or prevent DSB repair.^4,5^ For instance, Poly-ADP ribose polymerase 1 (PARP1) inhibitors (PARPi) preferentially kill BRCA mutant cancer cells and are approved to treat HRD cancers.^6^ However, only ∼60% patients respond to PARPi and drug resistance is a major problem.^6,7^ Thus, developing second-generation precision medicines that target HRD cancers while suppressing PARPi resistance is urgently needed.

In 2015, Polθ was identified as a potential cancer drug target with high potential for inducing synthetic lethality (SL) in HRD cancers.^8,9^ Polθ is a multifunctional DNA repair enzyme possessing an amino-terminal SF2 helicase (Polθ-hel), an unstructured central domain, and a carboxy-terminal DNA polymerase (Polθ-pol), which has been highly characterized.^10–14^ Polθ promotes DSB repair via microhomology-mediated DNA end-joining (MMEJ)—also known as theta-mediated end-joining (TMEJ)—by facilitating DNA synapsis and DNA extension of minimally paired 3′ single-strand DNA (ssDNA) overhangs generated from 5′-3′ resection of DSBs.^11,14,15^ Polθ also promotes translesion synthesis and DNA gap filling, and all of these functions require Polθ-pol activity.^11,15–17^ Suppression of Polθ induces SL in HRD cells, including breast, ovarian and pancreatic cancer cells.^8–19^ In contrast, Polθ null mice show no major phenotypes.^9^ Thus, Polθ is a nonessential gene and an ideal precision oncology drug target. This is in contrast to other DDR inhibitors which cause toxicity due to the essential function of their respective targets (i.e., ATR, WEE1, Chk1).^20^ Polθ is upregulated in the majority of breast tumors as well as ovarian and pancreatic cancers,^9,19,21,22^ and Polθ overexpression correlates with HRD and a poor outcome for cancer patients.^9,19,21,23^ Polθ also confers cellular resistance to PARPi, ionizing radiation and other DNA damaging agents.^9,13,17,24,25^

Currently, two Polθ-pol inhibitors (ART6043, ART4215) and four Polθ-hel inhibitors (GSK4524101, RP3647, MOMA-313, SYN818) have entered clinical trials for treating DNA repair deficient solid tumors as single agents and in combination with PARPi which demonstrates strong interest in Polθ as a promising cancer drug target. Here, we report the development of RTx-303, a small-molecule allosteric Polθ-pol inhibitor that exhibits 5.1 nM IC_50_, 88% oral bioavailability in mice, and a prolonged half-life along with its equipotent metabolite in plasma, liver microsomes and hepatocytes. RTx-303 exhibits higher cellular potency and tolerability than previously reported Polθ-pol inhibitors and strongly potentiates PARPi in *BRCA1* and *BRCA2* mutant xenografts.

## Results

### Structure-Based Development of RTx-284

Recent studies have demonstrated the potential of Polθ-pol inhibitors (Polθi) in BRCA mutant cells and tumor models which bind the same allosteric pocket. For example, prior studies revealed potent and selective inhibitors of Polθ-pol (ART558, ART812, ART899) (Figure 1).^18,26,27^ ART812 showed improved metabolic stability compared to ART558, but exhibited a relatively short half-life (T1/2) and limited exposure in rat.^18,27^

**Figure 1:**
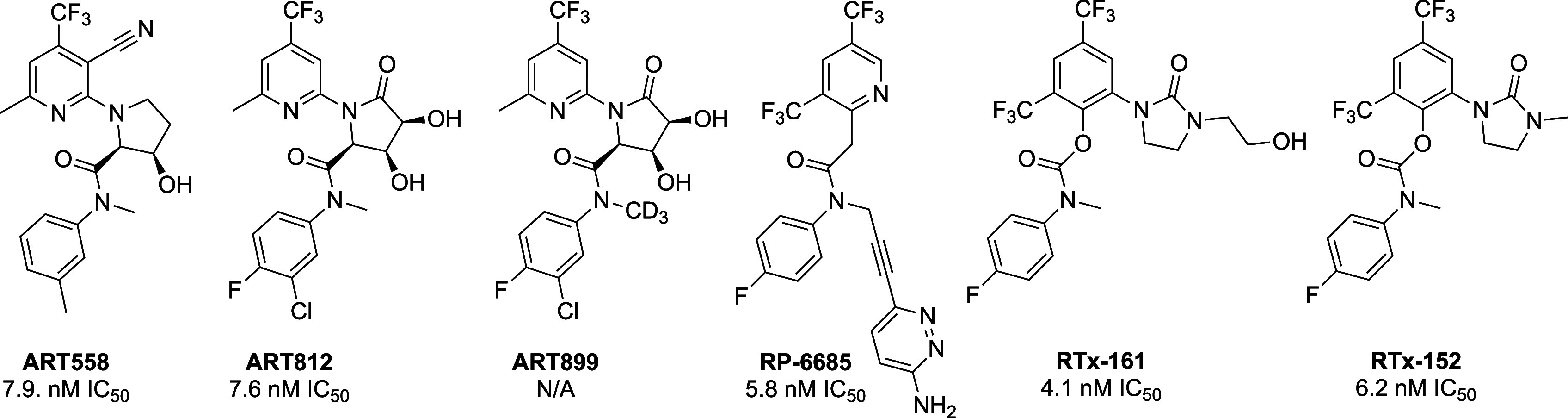
Representative Polθ-pol inhibitors. N/A, not available.

ART899 exhibited improved metabolic stability but required a relatively high dose (150 mg/kg, BID) for pharmacological activity in mice.^26^ Another recent report revealed a different Polθ-pol inhibitor (RP6685) (Figure 1).^28^ This compound showed a relatively short half-life in plasma and lost in vivo pharmacological activity in mice after 2 weeks, potentially due to induced metabolic inactivation.^28^ We recently developed a third Polθ-pol inhibitor series (RTx-161/*RT*x-152) that exhibited single-digit nanomolar potency, strong synergistic activity with PARPi in HRD cells, and trapped Polθ-pol onto DNA as its mechanism of action (Figure 1).^25^ This compound series, however, showed poor metabolic stability.^25^

RTx-152/161 were previously developed from a heterocyclic-based carbamate derivative (MC2800003) identified from a high-throughput screening campaign (Figure 2A). The poor liver microsome stability of RTx-152/161, however, prevented evaluation of in vivo efficacy. To improve the metabolic stability of this Polθi we employed X-ray crystallography and medicinal chemistry. We solved a high-resolution X-ray structure of Polθ-pol bound to a DNA primer-template and RTx-161 to a resolution of 3.31 Å, which revealed the ethanol side-chain occupying solvent exposed space outside the hydrophobic binding pocket (Figure 2B and Table S1). Within the allosteric binding pocket, RTx-161 engages in hydrophobic interactions with multiple surrounding residues and forms two hydrogen bonds with R2347 and R2419 (Figure 2C). Structural comparisons of Polθ-pol bound to RTx-161 and RTx-152 revealed significant differences in their binding interactions. For example, the ethanol side chain of RTx-161 pulls the inhibitor toward the M-helix of the binding pocket, forming additional hydrophobic interactions with V2351 and V2358 that are absent in the RTx-152 complex (Figure S1).

**Figure 2:**
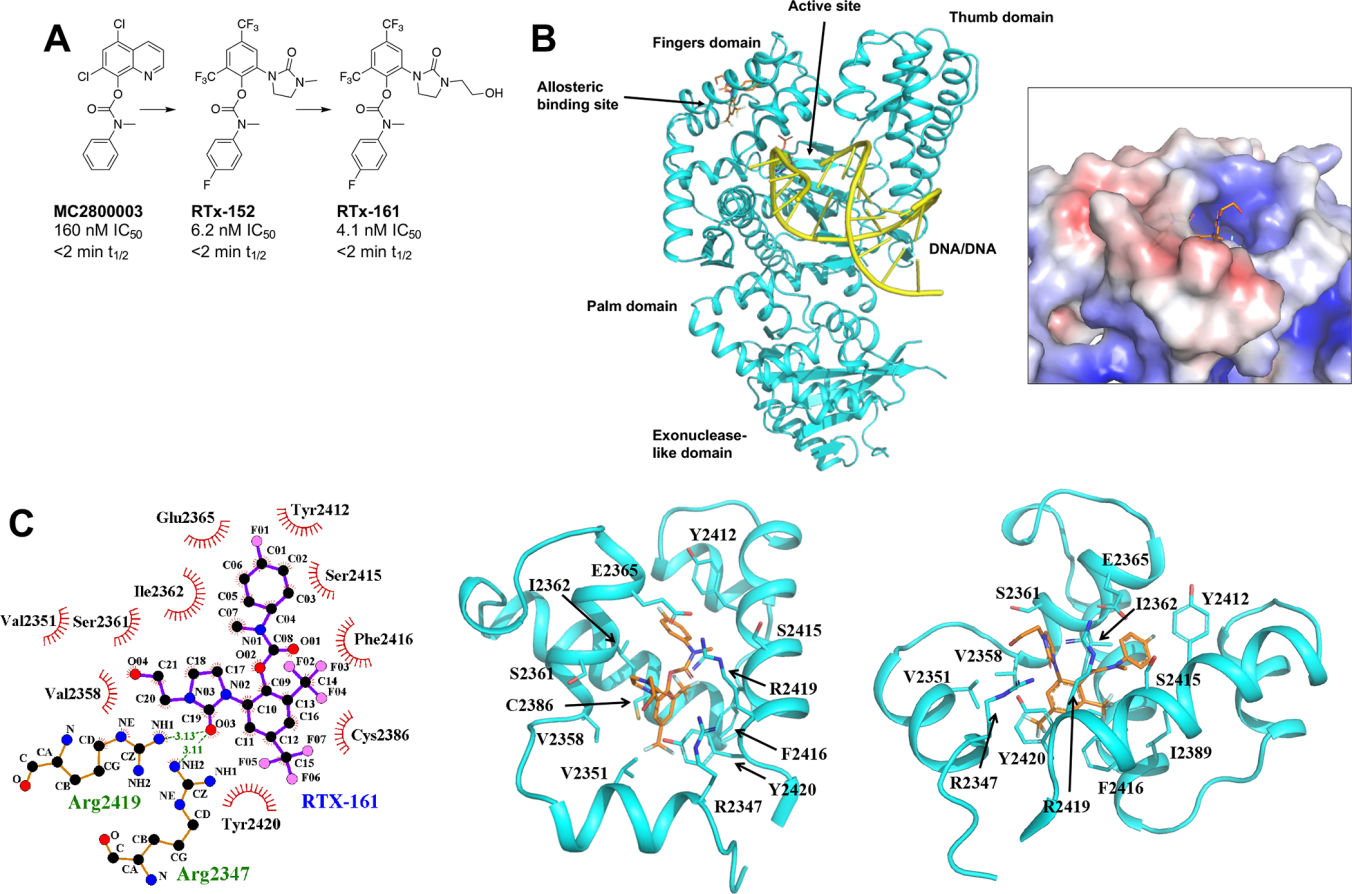
Co-crystal structure of Polθ-pol:DNA:ddGTP:RTx-161 complex. A. Structures of previous Polθi. B. Overall crystal structure of Polθ-pol:DNA:ddGTP:RTx-161 complex (left). The electrostatic surface representation of Polθ-pol around the allosteric inhibitor binding site (right). Positively charged potential is in blue, negative potential in red, and neutral in white. RTx-161 is represented as sticks. C. The left image shows the 2D ligand interaction plot detailing interactions between inhibitor RTx-161 and the surrounding Polθ-pol residues at the inhibitor-binding pocket. Hydrophobic contacts are shown by the red radiating symbols while hydrogen bonds are showcased by green dashed lines. The center and right images are detailed structures showing the 3D positions of the Polθ-pol residues shown on the left within the allosteric binding site in relation to RTX-161.

Based on the Polθ-pol:DNA:RTx-161 structure, RTx-161 analogs were synthesized with different polar side-chains and screened for in vitro IC_50_, mouse liver microsome stability, and IC_50_ against BRCA2+/+ and BRCA2–/– HCT116 cells to identify derivatives with high potency against Polθ-pol and BRCA2–/– cells, and improved metabolic stability. The *N*-methyl within the carbamate linker was hypothesized to confer metabolic liability. Thus, some derivatives were synthesized with deuterium replacement of the hydrogens within the *N*-methyl motif to improve metabolic stability. Representative analogs are illustrated in Table 1. Clonogenic survival assays demonstrated that RTx-284 and RTx-283 exhibited a > 10-fold lower IC_50_ in BRCA2–/– HCT116 cells compared to RTx-161 and showed >100-fold selectivity against BRCA2–/– versus BRCA+/+ HCT116 cells (Table 1). These analogs also exhibited single-digit nanomolar IC_50_ against recombinant Polθ-pol using an *in vitro* fluorescent-based DNA synthesis assay (Table 1). RTx-283 and RTx-284 respectively exhibited 44.8 and 8.0 min half-life (T1/2) in mouse liver microsomes which were significant improvements in metabolic stability compared to RTx-161 (T1/2 < 2 min) (Table 1). RTx-283 and RTx-284 showed significantly longer T1/2 in human and rat liver microsomes, and mouse hepatocytes (Table 2). Derivatives of RTx-283 and RTx-284 lacking deuterium atoms (RTx-290, RTx-182) exhibited nearly identical T1/2 in mouse liver microsomes (Table 1).

**Table 1:**
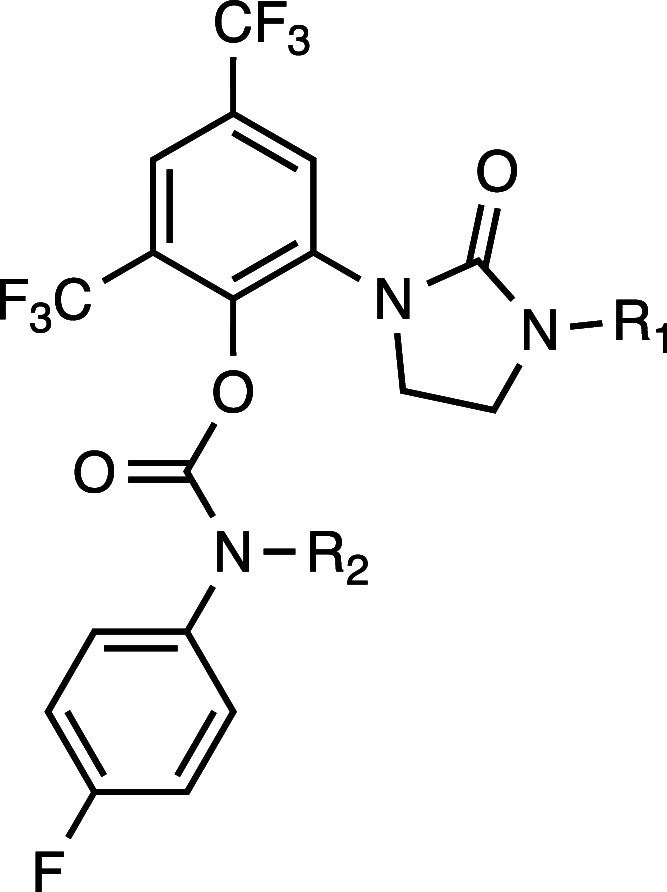

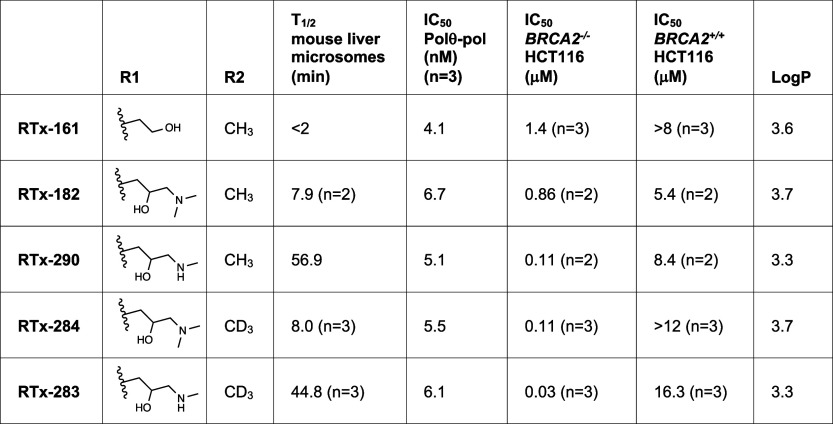
Dimethyl and Monomethyl Side-Chains Improve Metabolic Stability and Cellular Potency

**Table 2:**
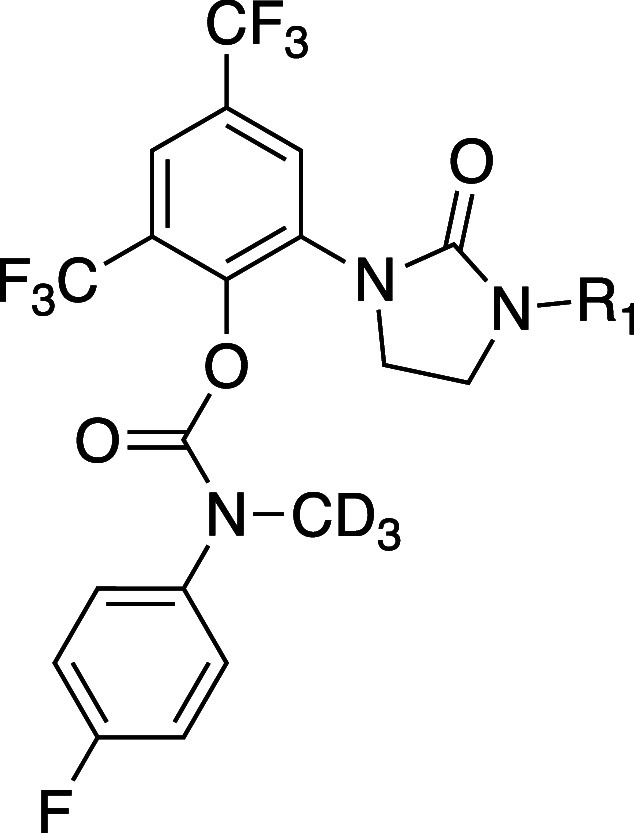

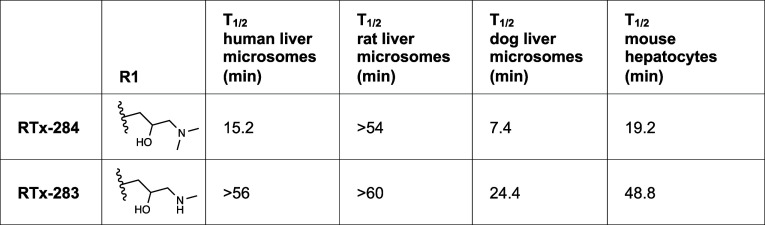
Metabolic Stability of Analogs with *N*-Methyl Deuteration

Next, we analyzed the PK of RTx-283, RTx-284 and their respective derivatives lacking deuterium (RTx-290, RTx-182) in CD-1 mice. RTx-284 exhibited 100% oral bioavailability and significantly higher exposure than the other closely related analogs (Table 3, top). These data demonstrated that the dimethylamine motif within RTx-284 and RTx-182 strongly contributes to their intestinal absorption, and that the deuterium atoms enhance in vivo exposure. In vitro ADME studies revealed that the dimethylamine containing analogs (RTx-284, RTx-182) exhibit lower plasma protein binding than the derivatives containing monomethylamine (RTx-283, RTx-290) (Table 3, top). Further in vitro ADME studies demonstrated that RTx-284 and RTx-283 exhibit >200 μM solubility and >3 h T1/2 in PBS, plasma, and simulated intestinal and gastric fluids (Figure S2).

**Table 3:**
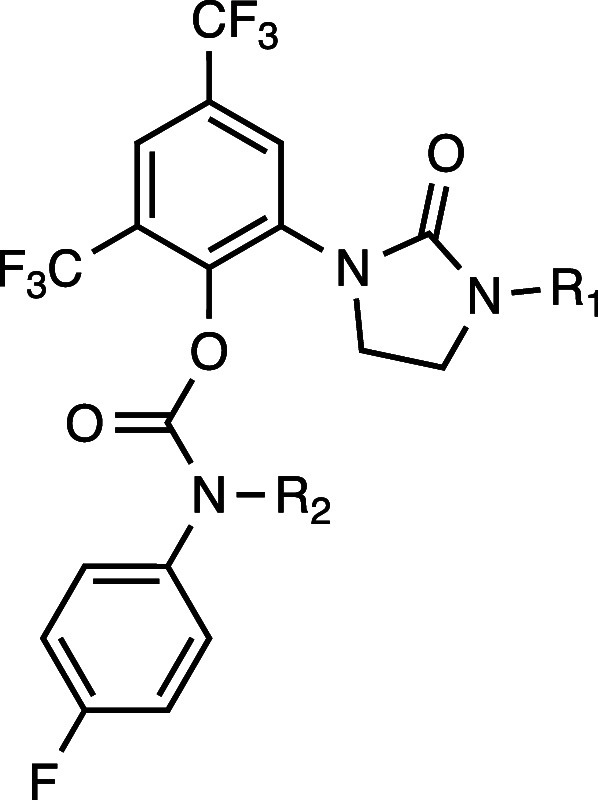

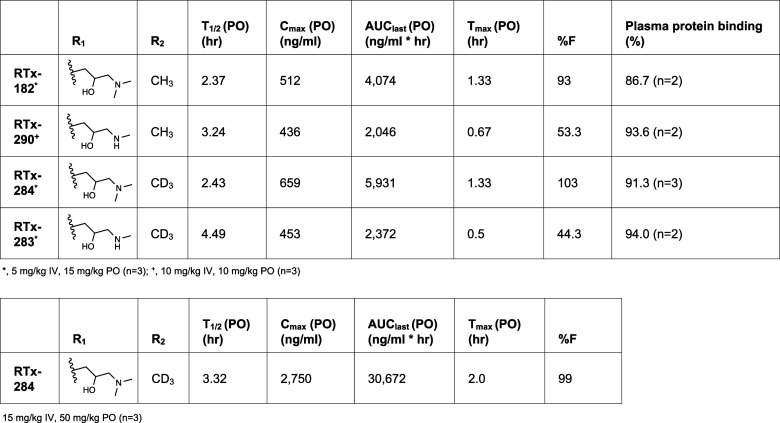
Dimethylamine Side-Chain Increases Oral Bioavailability

To elucidate the binding mode of RTx-284, we solved the X-ray structure of the Polθ-pol:DNA complex bound to RTx-302, a pure enantiomer of RTx-284 which is a racemic mixture (Figure 3A). As expected, the dimethylamine side-chain of RTx-302 is solvent exposed, while the remainder of the inhibitor resides within a slightly positively charged, enclosed allosteric binding pocket (Figure 3B). Within this pocket, RTx-302 engages in extensive hydrophobic interactions with surrounding residues and forms three hydrogen bonds with R2347, R2419, and Y2420 (Figure 3C). Close comparison of the RTx-161 and RTx-302 bound structures shows that the heterocyclic ring orientation modulates the binding efficiency of these inhibitors. For instance, a unique hydrogen bond between Y2420 and the oxygen atom on the 5-member heterocyclic ring of RTx-302 is observed, and the heterocyclic ring of RTx-302 is positioned to form hydrogen bonds with both R2347 and Y2420 (Figure S3).

**Figure 3:**
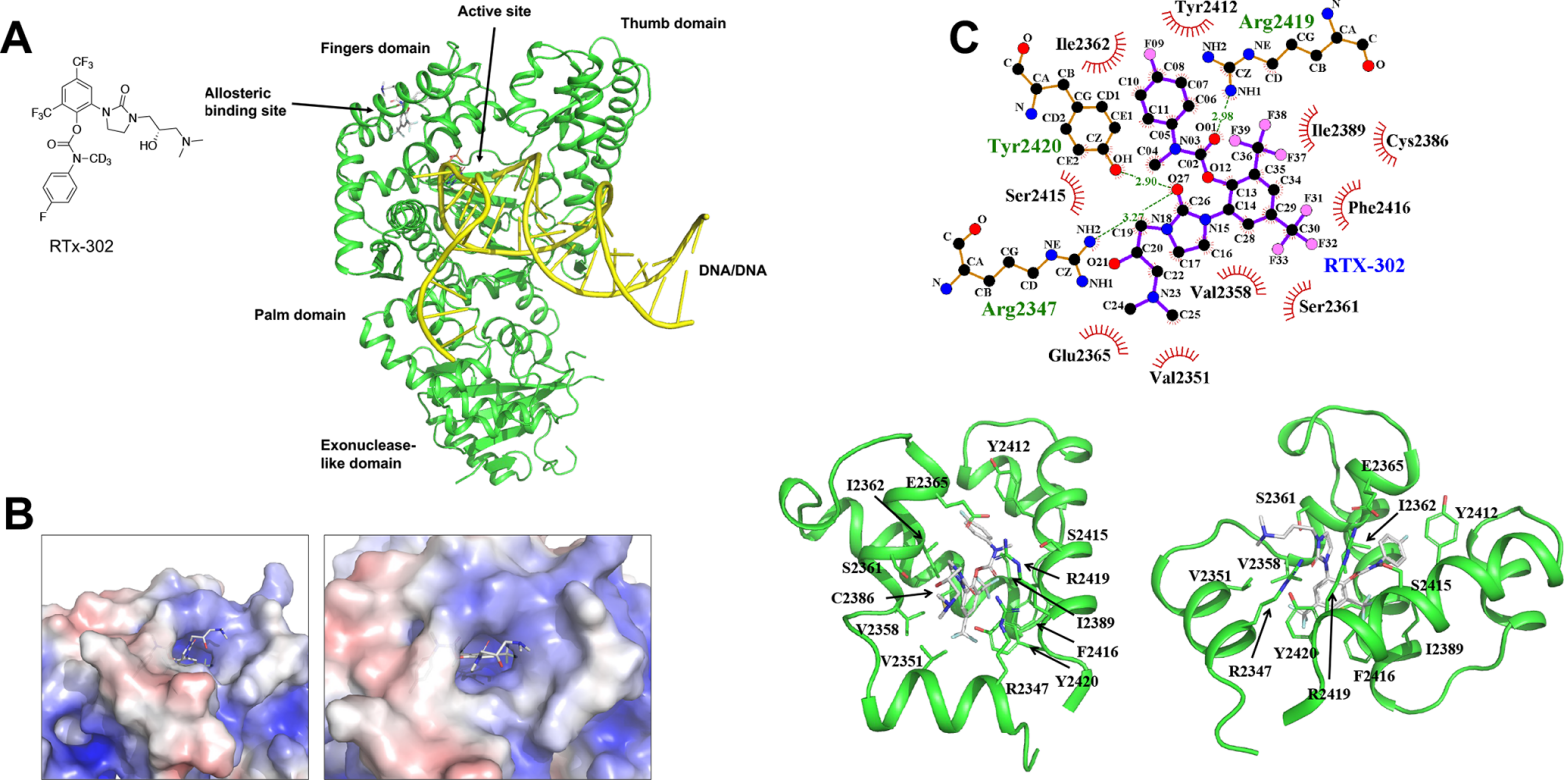
Co-crystal structure of Polθ:DNA:ddGTP:RTx-302 complex. A. Overall crystal structure of Polθ:DNA:ddGTP:RTx-302 complex. B. The electrostatic surface representation of Polθ-pol around the allosteric inhibitor binding site. Positively charged potential is in blue, negative potential in red, and neutral in white. RTx-302 is represented as sticks. C. The top image shows a 2D ligand interaction plot detailing interactions between RTx-302 and the surrounding allosteric pocket of Polθ-pol. Hydrophobic contacts are shown by the red radiating symbols while hydrogen bonds are showcased by green dashed lines. The bottom left and right images are detailed structures showing the 3D positions of the Polθ-pol residues shown on the top within the allosteric binding site in relation to RTx-302.

### Characterization of RTx-284 Metabolism and PK

Dimethylamine side-chains are frequently found in FDA-approved drugs and therefore contribute to drug-like properties and pharmacological activity.^29^ We hypothesized that the dimethylamine side-chain in RTx-284 is demethylated by liver enzymes to yield the equipotent RTx-283 monomethylamine metabolite (Figure 4A). Although RTx-283 exhibited a longer T1/2 in liver microsomes and hepatocytes than RTx-284 (Tables 1 and 2), it showed >2-fold lower oral bioavailability and in vivo exposure compared to RTx-284 (Table 3, top). Mass spectrometry analysis revealed that RTx-284 is metabolized into RTx-283 in mouse, human, rat and dog liver microsomes, and mouse hepatocytes (Figure 4B). Additional PK studies demonstrated that RTx-284 is metabolized into RTx-283 in mice and confirmed that RTx-284 exhibits high oral bioavailability (99%; Figure 4C,D and Table 3, bottom). The combined half-life and Cmax of the two equipotent Polθi (RTx-284 + RTx-283) following 50 mg/kg oral administration of RTx-284 was 4.1 h and 5.8 μM in plasma, respectively (Figure 4E).

**Figure 4:**
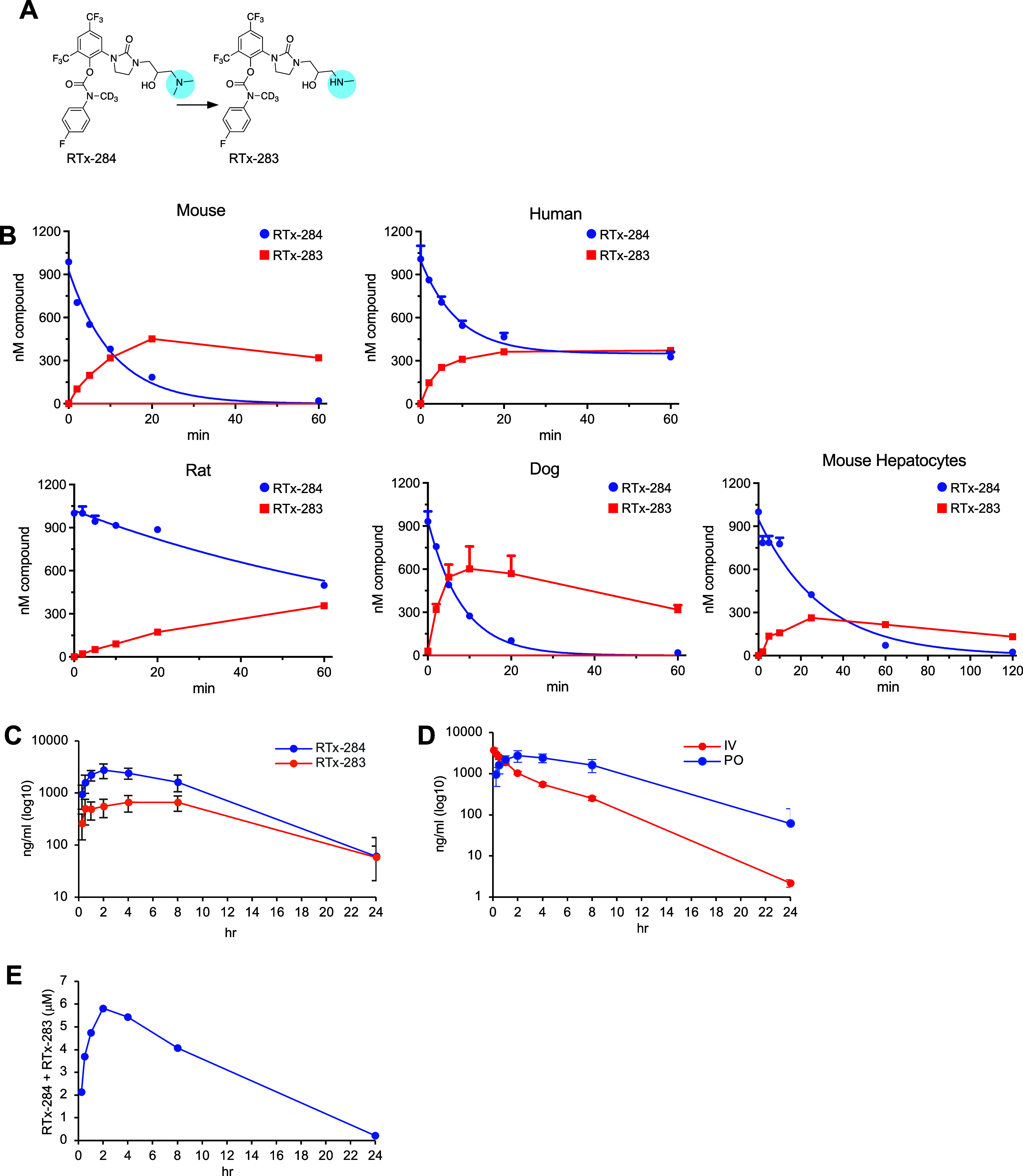
Metabolism and pharmacokinetics of RTx-284. A. Schematic of demethylation of RTx-284. B. Scatter plots showing the conversion of RTx-284 into RTx-283 over time in the indicated liver microsomes and hepatocytes. C. Scatter plot showing the concentration of RTx-284 and metabolite RTx-283 in plasma from CD-1 mice following PO administration of 50 mg/kg RTx-284. Data represent mean *n* = 3, ±SD. D. Scatter plot showing the concentration of RTx-284 in plasma from CD-1 mice following PO administration at 50 mg/kg (blue) and IV administration at 15 mg/kg (red). E. Scatter plot showing the concentration of the sum of RTx-284 and metabolite RTx-283 in plasma from CD-1 mice following PO administration of 50 mg/kg RTx-284.

### Characterization of RTx-284 Selectivity and Cellular Activity

RTx-284 demonstrated strong inhibition of Polθ-pol in vitro and no inhibition of other recombinant DNA polymerases, including the related A-family Polγ (Figure S4). As expected, RTx-284 showed selective killing of multiple HRD cells, with little to know effect in HR-proficient cells (Figure 5A), similar to its precursor, RTx-161^26^. RTx-284 also demonstrated strong synergy with multiple PARPi in MDA-MB-436 and PE01 cancer cell lines harboring BRCA1 and BRCA2 pathogenic mutations, respectively (Figure 5B–D). Using a previously reported MMEJ GFP reporter assay,^30^ we demonstrated that RTx-284 significantly inhibits Polθ MMEJ activity in cells, consistent with an on-target effect (Figure 5E). Consistent with the strong synergy between RTx-284 and olaparib, we observed that the RTx-284:olaparib combination induced significantly higher levels of the DNA damage marker γH2AX in DLD1 BRCA2–/– cells (Figure 5F) and in HCT116 *BRCA2–/–* xenografts versus RTx-284 or olaparib treatment alone (Figure S5). RTx-284 also promoted significantly higher levels of apoptosis in HCT116 BRCA2–/– cells versus HCT116 BRCA2+/+ cells, indicated by caspase positive cells (Figure 5G). Taken together, these data demonstrate that RTx-284 is a highly selective Polθ-pol inhibitor that targets HRD cells and synergizes with PARPi in HRD cells.

**Figure 5:**
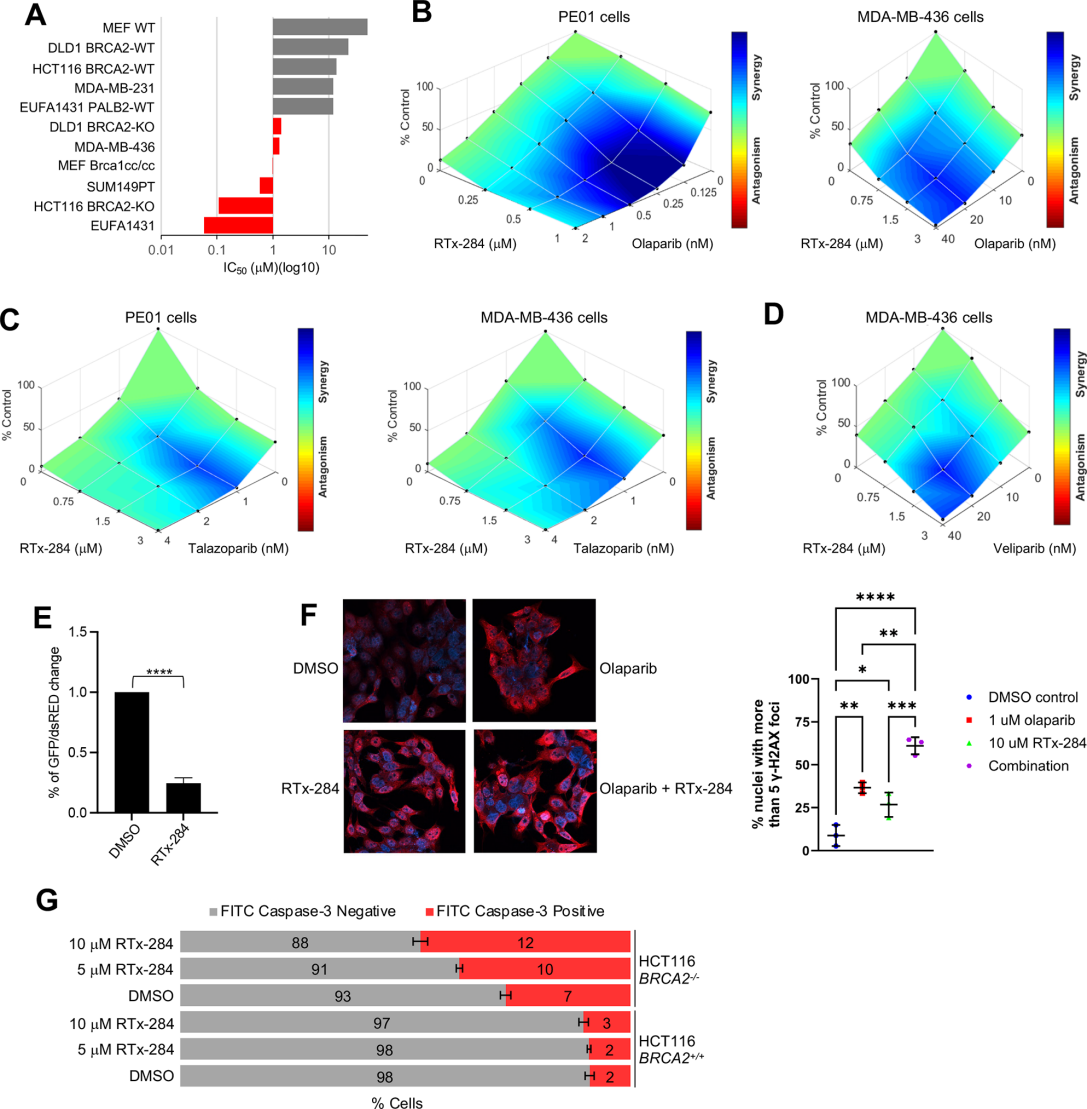
Cellular and in vitro activity of RTx-284. A. Bar plot showing the IC50 of RTx-284 in the indicated HR-proficient (gray) and HRD (red) cell lines. Data represent mean of at least 3 independent experiments performed in triplicate. B-D. Synergy plots created by Combenefit showing synergistic activity between RTx-284 and indicated PARPi in the indicated HRD cell lines. E. Bar plot showing that 10 μM RTx-284 suppresses the MMEJ GFP reporter. Data represent mean of 3 individual experiments performed in triplicate ±SEM. F. Dot plot showing increase of γH2AX following RTx-284:olaparib treatment in DLD1 BRCA2–/– cells. G. Bar plot showing that RTx-284 promotes an increase in caspase positive DLD1 BRCA2–/– cells. Data represent mean of 3 independent experiments performed in triplicate, ±SEM.

### Evaluation of RTx-284 Enantiomers

To determine the optimal drug lead, we purified the respective enantiomers of RTx-284 (RTx-302 and RTx-303) and compared their various properties. We confirmed that the dimethylamine in the respective enantiomers was metabolized into monomethylamine, similar to the RTx-284 racemate (Figure S6). The pure enantiomers and their monomethylamine metabolites (RTx-314, RTx-315) exhibited similar IC_50_ values and in vitro ADME parameters such as liver microsome stability and plasma protein binding (Table 4). Considering that the monomethylamine racemate RTx-283 exhibited approximately 2-fold lower oral bioavailability than the dimethylamine racemate derivative RTx-284, we focused on evaluating the pharmacokinetics of RTx-302 and RTx-303 and measured the concentration of their respective equipotent metabolites (RTx-314, RTx-315) formed in CD-1 mice. RTx-303 exhibited a higher Cmax, AUC and T1/2 compared to RTx-302 (Table 5, top). The metabolite of RTx-303 (RTx-315) also exhibited a higher Cmax and AUC than the RTx-302 metabolite (RTx-314) (Table 5, top). Overall, the avg free unbound exposure of RTx-303 was slightly higher than RTx-302, both with and without inclusion of the respective equipotent metabolites. Based on these studies, RTx-303 was chosen for further development. RTx-303 and its metabolite (RTx-315) exhibited significantly longer half-lives in rat, despite the reduced oral bioavailability of RTx-303%F, 58.6; Table 5, bottom). RTx-303 and RTx-315 also showed significantly reduced binding to rat plasma proteins (Table 5, bottom), and RTx-303 was most stable in human and rat hepatocytes (Table 6).

**Table 4:**
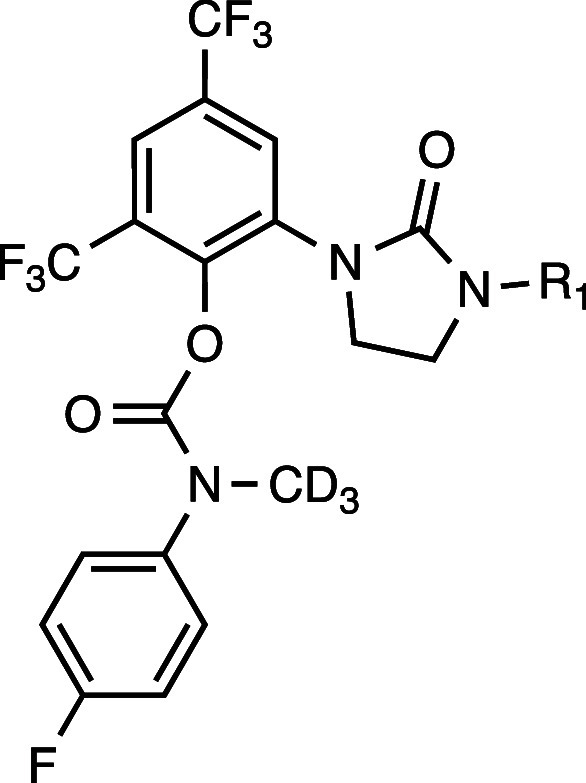

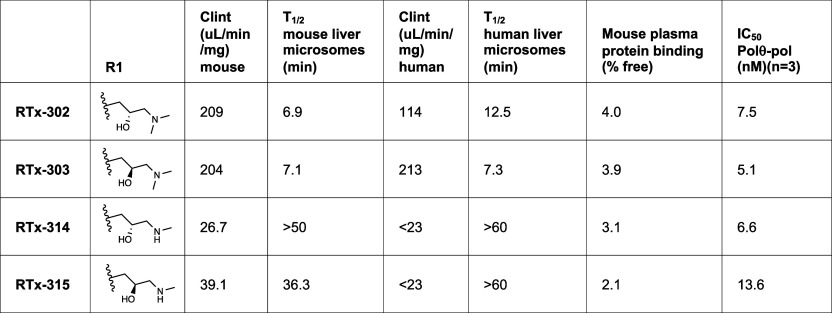
In Vitro ADME and Potency of Chiral Derivatives

**Table 5:**
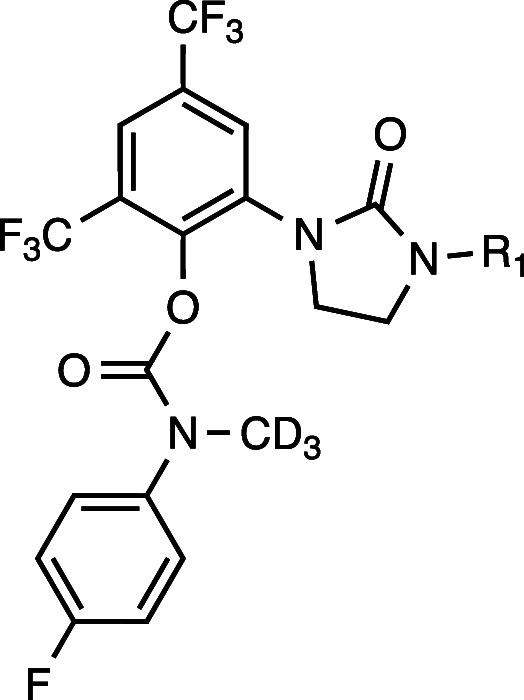

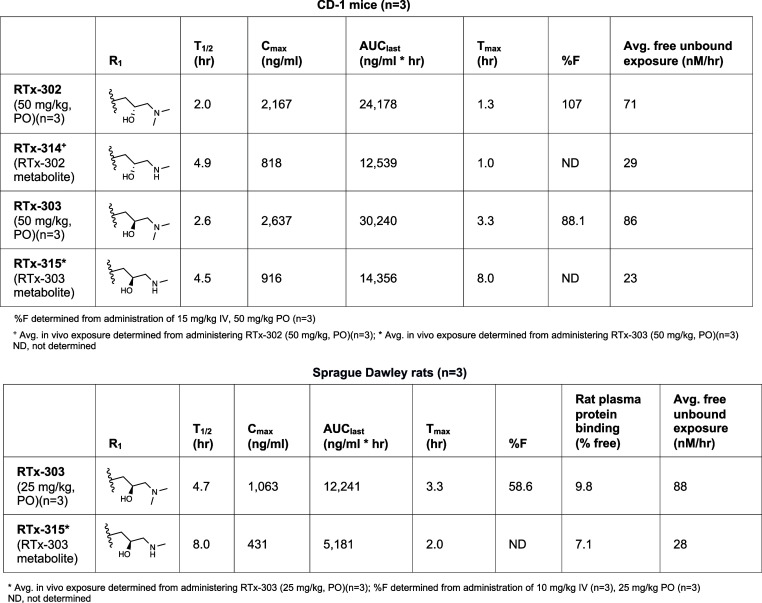
Pharmacokinetics of Pure Enantiomers and Equipotent Metabolites

**Table 6:**
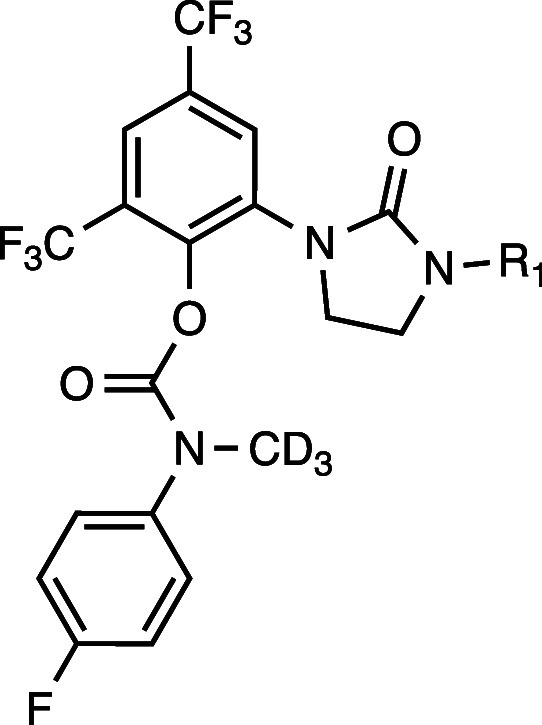

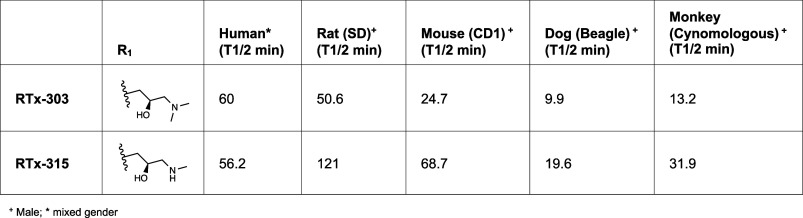
Stability of Pure Enantiomers in Hepatocytes

Similar to RTx-284, RTx-303 showed no significant inhibition of other eukaryotic DNA polymerases, including the related A-family Polγ (Figure S7). RTx-303 also showed no significant inhibition against a kinase panel (Figure S8). We next measured the cellular IC_50_ of RTx-303 versus previously reported Polθ-pol inhibitors to determine its relative cellular potency. Clonogenic survival assays demonstrated that RTx-303 exhibits significantly lower IC_50_ in HCT116 BRCA2–/– cells compared to Polθ-pol inhibitors RP-6685, ART558 and ART812 which bind to the same allosteric pocket as RTx-303 (Table 7, Figure S9).^18,28^ Further evaluation of RTx-303 in multiple cell lines showed that the inhibitor exhibits selective killing of HRD cells (Figure S10).

**Table 7:** Relative Potency of Polθ-Pol Inhibitors

Compound	IC_50_(nM) HCT116 *BR*CA2 –/–	IC_50_(nM) HCT116 *BR*CA2 +/+
RTx-303	81.2	>1280
RP-6685	403.2	>1280
ART812	454.1	>1280
ART558	1036	>1280

### RTx-303 Potentiates PARP Inhibitors in BRCA Mutant Cells and Xenografts

We confirmed that RTx-303 exhibits synergistic activity with PARPi *in vitro* in BRCA mutant cells, similar to RTx-284 (Figure S11). We next evaluated the ability of RTx-303 to potentiate olaparib in ID8 *Trp53–/–;Brca2–/–* mouse cells which are used as a model for ovarian cancer.^31^ ID8 *Trp53–/–;Brca2–/–* cells were sensitive to olaparib (2 μM IC_50_), potentiated the activity of olaparib as expected (Figure 6A; 0.12 μM IC_50_). ID8 *Trp53–/–;Brca2–/–* PARPi-resistant (PARP-R) cells were generated through serial treatment and passaging of ID8 *Trp53–/–;Brca2–/–* cells with progressively increasing concentrations of olaparib until resistance was established.^32^ The ID8 *Trp53–/–;Brca2–/–* PARPi-resistant cells exhibited a > 6-fold higher IC_50_ for olaparib (13.3 μM IC_50_) compared to the ID8 *Trp53–/–;Brca2–/–* PARPi-sensitive cells (2 μM IC_50_; Figure 6A). Notably, the addition of RTx-303 also potentiated olaparib in the ID8 *Trp53–/–;Brca2–/–* PARPi-resistant cells (Figure 6A). Hence, these data reveal that RTx-303 enhances the activity of olaparib in *BRCA2–/–* cells selected for preexisting PARPi resistance.

**Figure 6:**
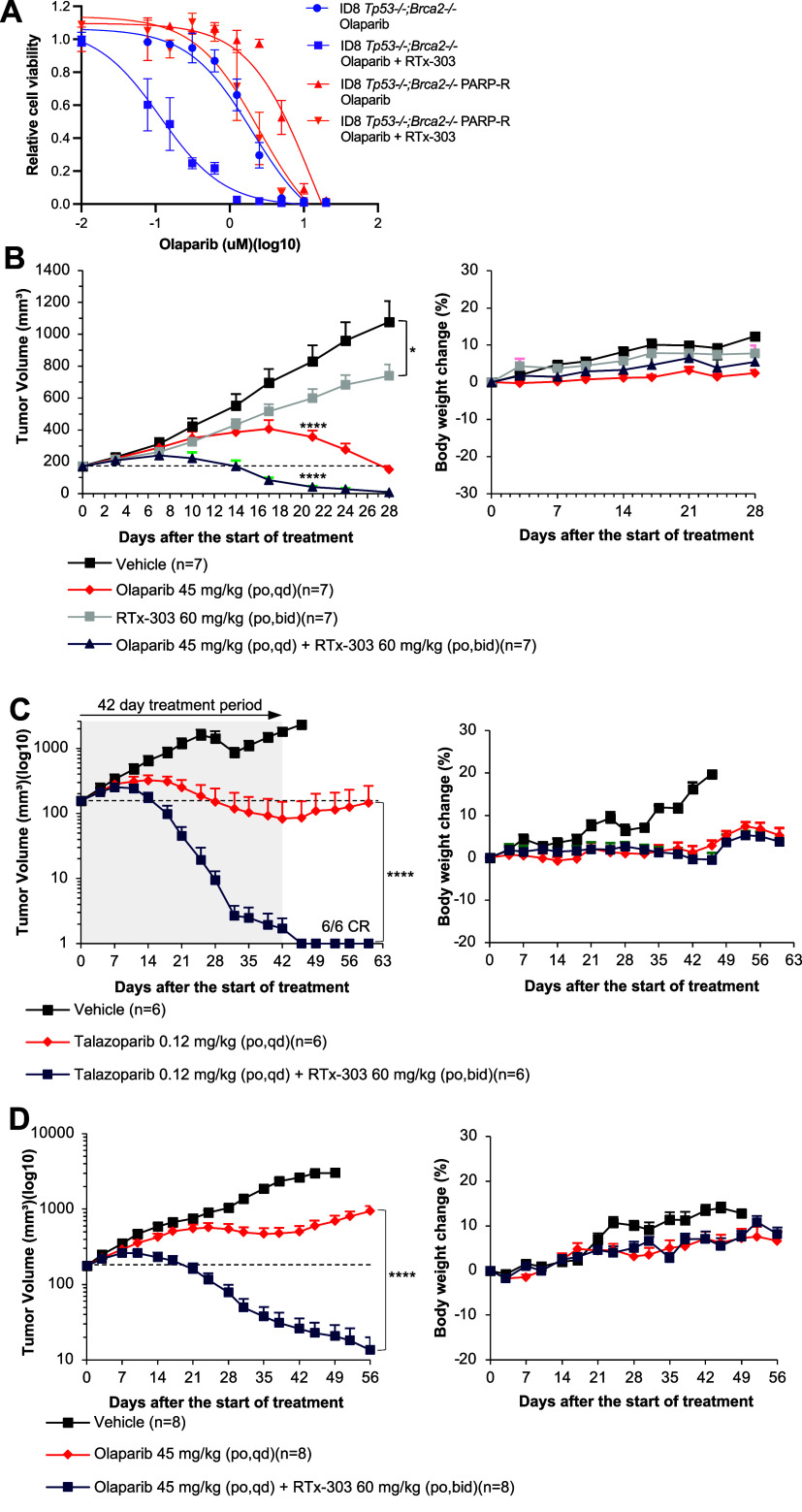
RTx-303 potentiates PARPi in BRCA mutant cells and xenografts. A. Scatter plot showing relative viability of the indicated ID8 cells treated with the indicated concentrations of olaparib with and without 5 μM RTx-303. Data represent mean of 3 independent experiments performed in triplicate, ±SEM. B. Scatter plot showing BRCA2 mutant BR-05–0566 PDX volumes following treatment with vehicle, 45 mg/kg olaparib (PO,QD), 60 mg/kg RTx-303 (PO,BID), and 45 mg/kg olaparib (PO,QD) with 60 mg/kg RTx-303 (PO,BID) (left). Scatter plot showing % body weight change (right). Data represent mean, *n* = 7 ±SEM. C. Scatter plot showing BRCA2 mutant BR-05–0568 PDX volumes following treatment with vehicle, 0.12 mg/kg talazoparib (PO,QD) with and without 60 mg/kg RTx-303 (PO,BID) (left). Scatter plot showing % body weight change. (right). Data represent mean, *n* = 6 ±SEM. D. Scatter plot showing MDA-MB-436 tumor volumes following treatment with vehicle, 45 mg/kg olaparib (PO,QD) with and without 60 mg/kg RTx-303 (PO,BID) (left). Scatter plot showing % body weight change (right). Data represent mean, *n* = 8 ±SEM. *p < 0.05, ***p < 0.001.

Next, the in vivo efficacy of RTx-303 was evaluated at 60 mg/kg (po, bid) as a single agent and in combination with olaparib against a *BRCA2* mutant breast cancer PDX model (BR-05–0566) in BALB/c mice. This dose was chosen based on the sum of the free unbound exposure of RTx-303 and its equipotent metabolite RTx-315 in mice which exceeds the IC_50_ of RTx-303 in *BRCA2–/–* cells (81.2 nM) (Table 5). The 60 mg/kg (bid) dose showed significant tumor growth inhibition as a single agent and potentiated olaparib activity (Figure 6B, left). Furthermore, no significant body weight loss was observed (Figure 6B, right). RTx-303 was next evaluated in combination with talazoparib, the most potent PARPi. RTx-303 was administered at 60 mg/kg (po, bid) in combination with 0.12 mg/kg (po, qd) talazoparib for 42 days in female BALB/c mice inoculated with different *BRCA2* mutant breast cancer PDX model (BR-05–0568). The combination treatment resulted in complete elimination of all tumors (Figure 6C, left). In contrast, talazoparib single agent treatment only moderately regressed tumors, and the average tumor volume increased immediately following termination of talazoparib treatment. No significant body weight loss was observed despite the potential for talazoparib to induce toxicity (Figure 6C, right).

RTx-303 additionally enhanced olaparib activity in the *BRCA1* mutant triple negative breast cancer (TNBC) MDA-MB-436 cell derived xenograft model. Here, tumors showed a temporary response to olaparib between days 24 and 42, then 100% of tumors became resistant to olaparib and steadily increased in volume until the end of the study (Figure 6D, left). In contrast, combining olaparib with 60 mg/kg RTx-303 (po,bid) resulted in rapid tumor regression. These data suggest that Polθ-pol inhibition via RTx-303 treatment prevents acquired PARPi resistance. Furthermore, no significant body weight loss was observed for all three cohorts (Figure 6D, right). Moreover, no significant body weight loss or unhealthy signs were observed when RTx-303 was administered to mice at significantly higher doses (200 mg/kg, bid), demonstrating higher tolerability than previously published Polθ-pol inhibitors (Figure S12).^18,28^ Taken together, these data demonstrate that PARPi combination with RTx-303 exhibits more rapid and sustained tumor regression in BRCA mutant tumors compared to PARPi single agent activity.

## Discussion and Conclusions

Polθ is an exciting precision oncology drug target due to its essentiality in HRD cells and ability to promote resistance to PARPi and other DNA damaging cancer therapies. Although Polθ-pol inhibitors have been previously reported, these compounds exhibited limited oral bioavailability, tolerability, metabolic stability, or pharmacological activity.^18,26,28^ Here, we report the development of RTx-303, a potent and selective Polθ-pol inhibitor that exhibits 88% oral bioavailability in mice, >200 μM solubility, and a prolonged half-life in liver microsomes and hepatocytes along with its equipotent metabolite, RTx-315. RTx-303 strongly potentiates olaparib and talazoparib in BRCA mutant xenografts and exhibits a favorable safety profile in mice. RTx-303 and its active metabolite RTx-315 also exhibit high stability in simulated intestinal and gastric fluids, plasma and PBS buffer. Notably, RTx-303 exhibited significantly higher potency in *BRCA2–/–* cancer cells compared to previously reported Polθ-pol inhibitors, and showed higher tolerability in mice. Overall, the superior potency, optimized drug-like properties, robust pharmacological activity, and favorable tolerability of RTx-303 warrant further development as a drug candidate.

Currently, there are multiple Polθ inhibitors targeting the polymerase and helicase domain in clinical trials for treating HRD cancers as single agents and in combination with PARPi. These clinical trials may reveal whether there are significant differences in the respective safety profiles of Polθ-hel inhibitors versus Polθ-pol inhibitors considering their significant differences in chemical structures.

## Experimental Section

### Protein Purification for X-ray Crystallization

A codon-optimized Polθ-pol sequence for bacterial expression was ordered from Thermo Fisher, with five flexible loop regions replaced by glycine-serine spacers of varying lengths, tailored to the distance between the deleted loops, resulting in PolθΔL as previously described.^10,25^ The resulting PolθΔL construct was cloned into a vector containing a N-terminal 6xHis tag, SUMO tag followed by the PreScission protease cleavage site as described.^10,25^ Protein expression and purification was performed by following the protocol as previously reported.^25^ Briefly, *E. coli* BL21­(DE3) cells transformed with PolθΔL clone were cultured at 37 °C in LB medium until the optical density at 600 nm (OD600) reached 0.3–0.4. The culture temperature was lowered to 16 °C, and the cells continued growing until OD600 reached 0.7–0.9. Protein expression was induced with 0.1 mM isopropyl β-D-thiogalactopyranoside, followed by overnight incubation at 16 °C. The cells were harvested by centrifugation and resuspended in Buffer L (50 mM HEPES, pH 8.0, 500 mM NaCl, 0.005% (v/v) Igepal CA-630, and 0.5 mM TCEP). Lysis was achieved by sonication in the presence of DNase I (100 μg/mL), RNase A (100 μg/mL), 10 mM MgCl2, 2 mM CaCl2, and 1 mM phenylmethylsulfonyl fluoride. The lysate was centrifuged, and the supernatant was subjected to Ni-NTA agarose gravity-flow chromatography. The resin was washed with Buffer W (50 mM HEPES, pH 8.0, 500 mM NaCl, 0.005% (v/v) Igepal CA-630, 0.5 mM TCEP, and 10 mM imidazole). The fusion protein was cleaved from the 6xHis tag by overnight incubation at 4 °C with Buffer L containing 1.25% (v/v) PreScission Protease. The cleaved PolθΔL protein was eluted with Buffer L, concentrated to approximately 1 mL, and treated with 0.2% Benzonase and 5 mM MgCl2 to remove bound DNA. The protein was further purified using a HiTrap Heparin column followed by size exclusion chromatography with a Superdex 200 column (GE Healthcare). The final PolθΔL protein was concentrated to 37.74 mg/mL in a buffer containing 150 mM ammonium acetate, 150 mM KCl, 40 mM Tris-HCl (pH 8.0), 2.5 mM TCEP, and 1% (v/v) glycerol. The purified protein was aliquoted and stored at −80 °C.

### Co-Crystallization

The crystallization conditions for PolθΔL in complex with primer/template dsDNA, ddGTP, and RTx-161 or RTx-302 were optimized using hanging-drop vapor diffusion. Initial conditions were derived from previous screening of PolθΔL complexes with RTx-152. For crystallization, PolθΔL was mixed at 2.5 mg/mL with 50 μM of a DNA duplex (primer: 5′-CGACGTCGCAGCGC-3′; template: 5′-GCGAGACTCCGCGCTGCGACGTCG-3′; synthesized and HPLC-purified by IDT) and either 170 μM RTx-161 or RTx-302 (100% DMSO), in the presence of 1 mM ddGTP, 300 μM sucrose monolaurate, 1 mM MgCl2, and 20 mM spermine tetrahydrochloride.

Crystals for data collection of PolθΔL with RTx-161 were obtained by incubating 2 μL of the protein solution with 1 μL of a crystallization solution composed of 0.1 M Bis-Tris Propane (pH 7.5), 18% (w/v) PEG 3350, and 0.29 M sodium tartrate at 4 °C in a hanging drop tray over a 900 μL reservoir of the same crystallization solution. Crystals were cryoprotected by transferring them into the mother solution, supplemented with 20% glycerol, before flash-cooling in liquid nitrogen. Crystals of PolθΔL complexed with RTx-302 were obtained by incubating 1.5 μL of the protein solution with 1 μL of a crystallization solution containing 0.1 M Bis-Tris Propane (pH 7.5), 16% (w/v) PEG 3350, and 0.23 M sodium citrate at 4 °C in a hanging drop tray over a 900 μL reservoir of the same crystallization solution. Cryoprotection was achieved by transferring the crystals into the mother liquor supplemented with 20% ethylene glycol before flash-cooling in liquid nitrogen.

### Data Collection and Structural Determination

Diffraction data for the PolθΔL complex with RTx-161 were collected at beamline 5.0.3 of the Advanced Light Source at Lawrence Berkeley National Laboratory, Alameda County. A complete data set was obtained using BOS and subsequently processed with DIALS.^33^ Molecular replacement was performed using the Phaser-MR program from the PHENIX package. Model building and refinement were carried out with COOT and Phenix, using simulated annealing and iterative refinement.^34^ Ligands were generated with the eLBOW program in Phenix based on their SMILES representations, and any geometric inaccuracies were corrected using REEL.^35^ The initial molecular replacement model was the PolθΔL structure (PDB ID: 8GD7), with the inhibitor removed. Phase improvement was achieved through cyclic model building and refinement, resulting in a well-fitted model. The final structure of the PolθΔL complex with RTx-161 was resolved to a resolution of 3.31 Å (Table 1).

Diffraction data for the PolθΔL complex with RTx-302 were collected at beamline 8.2.2 of the Advanced Light Source at Lawrence Berkeley National Laboratory, Alameda County. As with RTx-161, data acquisition was performed using BOS and processed with DIALS.^33^ Molecular replacement was conducted using the Phaser-MR program from the PHENIX suite, followed by iterative model building and refinement with COOT and Phenix.^34^ Ligand geometries were prepared using the eLBOW program in Phenix, and any geometric inaccuracies were corrected using REEL.^35^ The PolθΔL structure (PDB ID: 8GD7), with the inhibitor removed, was used as the molecular replacement search model. Phases were iteratively improved through cyclic model building and refinement, yielding a high-quality final structure. The PolθΔL complex with RTx-302 was solved at a resolution of 2.43 Å (Table 1).

### Cell Lines

DLD1 BRCA2 −/– and DLD1 Parental were obtained from Horizon Discovery, Waterbeach, UK. HCT 116 BRCA2 −/– and HCT 116 Parental were obtained from Cancertools, London, UK. MEF BRCA1 −/– (CC), MEF Parental (Wildtype) and SUM149T were a kind gift from Dr. Neil Johnson (Fox chase Cancer Center). MDA 436 BRCA1 mut and MDA 231 (wildtype control for MDA 436) cells were obtained from ATCC, Manassas, VA. SKOV3 BRCA2 −/– and SKOV3 Parental were generated by CRISPR knockout cell line by using GenCRISPR gene editing technology (GenScript). SNU-601 cells was obtained from Cytion, Sioux Falls, SD. PEO1 was a kind gift from Sharon Cantor (University of Massachusetts). OVCAR8 was a kind gift from Sergey Karakashev (Temple University). DLD1 BRCA2 −/–, DLD1 Parental, PEO1, SNU-601, MDA 436 BRCA1 mut and MDA 231 were cultured in RPMI (GIBCO) supplemented with 10% fetal bovine serum (Cytiva), 2 mM l-glutamine (GIBCO), nonessential amino acids (GIBCO), and 1% penicillin/streptomycin (GIBCO). HCT 116 BRCA2 −/–, HCT 116 Parental, MEF BRCA1 −/– and MEF Parental, were cultured in DMEM (GIBCO) supplemented with 10% fetal bovine serum (Cytiva), 2 mM l-glutamine (GIBCO), nonessential amino acids (GIBCO), and 1% penicillin/streptomycin (GIBCO). SUM149T was cultured in Ham’s F-12 medium (GIBCO) with 10% fetal bovine serum (Cytiva), 2 mM l-glutamine (GIBCO), nonessential amino acids (GIBCO), and 1% penicillin/streptomycin (GIBCO). SKOV3 Parental and BRCA2 −/– were cultured in McCoys 5A medium (ATCC) supplemented with 10% fetal bovine serum (Cytiva), 2 mM l-glutamine (GIBCO), nonessential amino acids (GIBCO), and 1% penicillin/streptomycin (GIBCO)­U2OS cells with MMEJ reporter (EJ2-GFP) was a kind gift from Dr. Jeremy Stark (City of Hope) and were generated and described in prior studies.^36^ They were cultured in Dulbecco’s Modified Eagle Medium (GIBCO) supplemented with 15% fetal bovine serum (Cytiva), 2 mM l-glutamine (Sigma) and 1% penicillin/streptomycin (Sigma). Murine ovarian cancer cell line ID8 Trp53–/–;Brca2–/– was cultured in high-glucose DMEM supplemented with 1% penicillin/streptomycin, 4% fetal bovine serum, and 1% ITS (insulin-transferrin-selenium) as previously described.^31^ The olaparib resistant ID8 ID8 Trp53–/–;Brca2–/– cells were a gift from Dr. Bitler. The resistant cell line was developed from the parental ID8 Trp53–/–;Brca2–/– cell line by continuous exposure of the cells to progressively increasing concentrations of olaparib (ranging from 10 μM to 80 μM) in a stepwise manner as described.^32^

### MTT Assay

Cells were seeded on a 96-well plate at 1 × 10^4^ cells/well and treated with olaparib, RTx-284 or a combination. Each sample had three replicates. The medium was replaced every 2 days. Cell viability was assessed after 5-day treatment by the WST-1 assay (Sigma, Cat. # 5015944001) according to the manufacturer instructions.

### Colony Survival Assays

Colony survival assays were performed on 24-well plates. 800 cells/well of HCT, DLD1- BRCA2 −/–, PALB2 −/–, MEF BRCA1 cm^3^/cc, PEO1, SNU-601, SKOV3- BRCA2 −/–, OVCAR8 cells and 200 cells/well of HCT, DLD1, MEF, SKOV3 wildtype cells were plated. For MDA pair, 500 cells/well of MDA436 and 100 cells of MDA231 were plated. Cells were treated with the compound 24 h after plating and the medium was replaced every two or 3 days until the colonies were ready for staining. Colonies were typically ready for staining in 10–12 days. For staining: Medium was removed from plates, and cells were rinsed with PBS. Fixation was carried out with Water:Ethanol:Acetic acid (5:4:1) for 30 min followed by staining of colonies with 0.5% crystal violet in Water:Ethanol (3:2) for 2 h at room temperature. The plates were rinsed with water and left for drying overnight at room temperature. Colonies were then counted manually, and response curves are shown as mean colony formation ± SEM. IC_50_ was calculated with GraphPad Prism using nonlinear regression analysis, inhibitor vs response (variable slope) equation.

### Fluorescent-Based IC_50_ DNA Synthesis Assay

The biochemical Polθ-pol IC_50_ assay was developed in a prior report.^25^ Briefly, 60 nM of the preannealed primer-template containing a 5′ Cy5 fluorophore-conjugated template strand (RP486-Cy5), a downstream complementary oligo conjugated with a 3′ Iowa BlackReg Dark quencher (RP343BHQRQ) and a primer strand (RP469D) was mixed with 50 μM 2’-deoxyribonucleoside triphosphates (dNTPs), 0.1 mg/mL bovine serum albumin (BSA), 0.01% NP-40, 10% glycerol, 1 mM dithiothreitol (DTT), 10 mM MgCl_2_, 25 mM TrisHCl pH 7.8 in the presence of 2.5% DMSO with or without various concentrations (7-point dilution series) of Polθ-pol small-molecule inhibitors at 37 °C in a volume of 40 μL. The reactions were initiated by the addition of 5 nM of purified recombinant human Polθ-pol (comprising amino acid residues 1792–2590). The reactions were terminated by the addition of 20 mM EDTA after 18 min, and the Cy5 fluorescence intensity was measured using a CLARIOstar (BMG Labtech) plate reader. Reactions were performed in triplicate and the % inhibition at each concentration of the respective compound was based on the mean. The IC_50_ of each compound represents the average concentration of compound that resulted in 50% inhibition of Polθ-pol enzymatic activity which was determined from a scatter plot (% inhibition versus compound concentration) curve generated by GraphPad Prism 9 software for each compound inhibition data set.

## DNA

All the oligonucleotides were purchased from IDT. The sequences of the oligonucleotides (5′-3′) are listed below: RP343BHQRQ CTAAGCTCACAGTG/3IAbRQSp/; RP469D CTGTCCTGCATGATG; RP486-Cy5/5Cy5/CACTGTGAGCTTAGTCACATTTCATCATGCAGGACAG.

### Immunofluorescence

Immunofluorescence of γH2AX: Cells were plated on 6-well plates with glass-coverslips and treated with RTX-284 one day after plating. Four days after treatment, cells were fixed with 4% (v/v) paraformaldehyde for 20 min at 4 °C, washed with PBS, permeabilized with 0.5% (v/v) Triton X for 10 min and blocked with PBS containing 3% BSA. Cells were incubated with primary antibody (rabbit antigamma H2AX (p Ser139) antibody, Bethyl Lab #A700–053, 1:500 dilution in 1% BSA in PBS) overnight at 4 °C followed by 3x washes with PBS and then 1 h incubation with secondary antibody (Goat anti-Rabbit IgG (H+L) Secondary Antibody, DyLight 488 (Thermo #35552) 1:2000 dilution in 1% BSA in PBS). After 3x washing in PBS for 3 min, slides were mounted in 20 μl ProLong Antifade with DAPI (LifeTechnologies) to counterstain the nuclei. Cells were visualized and imaged using Nikon A1R Confocal microscope at a 63X objective magnification, and images were analyzed using ImageJ software. For quantification, >50 cells were counted for all conditions from three independent experiments.

### MMEJ GFP Reporter Assay

The GFP MMEJ reporter assay was performed as described. Briefly, U2OS cells carrying one copy of the previously described E2J-GFP MMEJ reporter cassette^30^ were sorted for GFP-positive cells followed by treatment with various concentrations of RTX-284 for 24 h before transfection. Pretreated cells were cotransfected with pCMV–3x- NLS-I-SceI or control vector pCMV-3x-NLS and dsRED-Mito cDNA (control for transfection efficiency) using lipofectamine 2000 (Invitrogen). 96 h post-transfection, GFP+ and dsRed+ frequencies were analyzed by flow cytometer (Facscanto, BD). Transfection efficiency was corrected using dsRed+ frequency and % MMEJ was calculated as the ratio of GFP+/dsRed+ cells. Data represent mean of 3 biological replicates ± SD.

### Apoptosis Assay

DLD1 BRCA2 −/–, DLD1 Parental cells were plated on 6-well plates and treated with RTX-284 1 day after plating. Medium was replaced every 2 days with RTX-161. Cells were harvested 6 days after plating for apoptosis assay using FITC active Caspase-3 apoptosis kit (BD biosciences, catalog # 550480) according to manufacturer’s instructions and analyzed by FACS with Celesta (Becton Dickinson)

### Statistical Analysis and Reproducibility

Data are expressed as mean ± SEM from at least 3 independent experiments with triplicates for each condition unless stated otherwise. Two-tailed unpaired *t* test was used for conducting comparison between two groups. Significance was assumed at *p* < 0.05. Asterisks in the figures indicate significance, *p < 0.05, **p < 0.01, ***p < 0.001. Statistically significant *p*-values and number of replicates are indicated in the figure legends.

### In Vitro ADME Assays

LCMS methods. Samples were analyzed with a Waters Acquity UPLC BEH C18 1.7 μM, 2.1 × 50 mm column at 40 °C where 5 μL samples were fractionated using a 3 min linear 5 to 95% acetonitrile gradient in 0.1% formic acid with a 0.55 mL/min flow rate. In tandem with the UPLC, compound concentrations were analyzed using a Waters Xevo TQ MS mass spectrometer in electrospray positive mode (source temperature 150 °C; desolvation temperature 550 °C; desolvation flow rate of 900 L/h). Compound electrospray mode, capillary voltages, cone voltages, MRMs and quantization were optimized and determined using Waters QuanOptimize software with propafenone as an internal standard. MRMs: RTX-284:570.27 > 155.07; RTX-283:556.27 > 127.04; Propafenone: 342.27 > 116.09). GraphPad Prism v 5.04 was used for nonlinear fitting of time course data to generate T1/2 values.

Mouse Plasma and PBS Stability. The stability of compounds at 1 μM were tested in duplicate at 37 °C in 500 μL of either 2.5% DMSO/97.5% mouse plasma (Innovative Research IGMSCD1PLANH50 ML; pooled plasma from CD1 mice; sodium heparin as anticoagulant; pH adjusted to 7.4) or 2.5% DMSO/97.5% phosphate-buffered saline (pH = 7.4 PBS: 136.9 mM NaCl; 2.68 mM KCl; 8.1 mM Na2HPO4; 1.47 mM KH2PO4; 0.9 mM CaCl2; 0.49 mM MgCl2). At 0, 30, 90, and 180 min time points, 50 μL aliquots were transferred to wells in a 96-well plate on ice containing 200 μL of ACN:water 85:15 containing the internal standard. After centrifugation (10 min; 4000 × *g*), supernatants were analyzed by LC/MS as described. Standard curves (0–1000 nM) were generated in PBS and plasma. Data were fit to a one-phase decay equation using GraphPad Prism 5.04 to generate T1/2 values.

Human Plasma and PBS Stability. The stability of compounds at 1 μM was tested in duplicate at 37 °C in 500 μL of either 2.5% DMSO/97.5%, human plasma (Innovative Research IPLAWBNH50 ML; pooled human plasma; sodium heparin as anticoagulant; pH adjusted to 7.4) or 2.5% DMSO/97.5% phosphate-buffered saline (pH = 7.4 PBS: 136.9 mM NaCl; 2.68 mM KCl; 8.1 mM Na2HPO4; 1.47 mM KH2PO4; 0.9 mM CaCl2; 0.49 mM MgCl_2_). At 0, 2, 5, 10, 30, 90, and 180 min time points, 50 μL aliquots were transferred to wells in a 96-well plate on ice containing 200 μL of ACN:water 85:15 containing the internal standard. After centrifugation (10 min; 4000 × *g*), supernatants were analyzed by LC/MS as described. Standard curves (0–1000 nM) were generated in PBS and plasma. Data were fit to a one-phase decay equation using GraphPad Prism 5.04 to generate T1/2 values.

Stability in PBS, SGF (simulated gastric fluid) and SIF (simulated intestinal fluid) assays. The stability of test compounds was determined in Dulbecco’s PBS (control solution; pH = 7.4; 136.9 mM NaCl; 2.68 mM KCl; 8.1 mM Na2HPO4; 1.47 mM KH2PO4; 0.9 mM CaCl_2_ and MgCl_2_, US Pharmacopeia, defined (US Pharmacopoeia 33–28NF; 2010) Simulated Gastric Fluid (SGF; 0.2% NaCl, 84 mM HCl, pH 1.2 with 3.2 mg/mL pepsin from porcine gastric mucosa), or US Pharmacopoeia-defined Simulated Intestinal Fluid (SIF; 50 mM potassium phosphate, pH 6.8; with 10 mg/mL pancreatin from porcine pancreas). Reactions were conducted in 96-well deep-well (1 mL) polypropylene plates in duplicate. 540 μL of prewarmed 1.11× PBS, SGF or SIF was added to wells of the prewarmed plate in the Labnet Vortemp incubator at 37 °C. Reactions were initiated by adding 60 μL of a 50 μM test compound in water (final concentration of 5 μM) to test wells. Samples were mixed and incubated with gentle shaking. At 0, 30, 90, 210 min, 75 μL aliquots were transferred to a stop plate (96-well plate with 300 μL of ACN:water 1:1 with 0.05 μM propafenone) on ice. After all time points, the stop plate was centrifuged 10 min at 2270 × *g* in a Beckman Coulter Allegra 6R centrifuge and 170 μL of the supernatants were transferred to a Waters Acquity UPLC 700 μL 96-well sample plate and sealed with a cap mat. After centrifugation (10 min; 4000 × *g*), supernatants were analyzed by LC/MS as described. Standard curves (0–1000 nM) were generated in PBS and plasma. Data were fit to a one-phase decay equation using GraphPad Prism 5.04 to generate T1/2 values.

Hepatocyte stability assays. Cryopreserved mouse hepatocytes (24 donors, male CD1 mice; Xenotech product M1000.H15+) or human hepatocytes (100 donors; mixed gender; Xenotech product HCP100.H15) were rapidly thawed, diluted with Optithaw (Xenotech product K8500) and centrifuged 5 min at 100 × *g*. The resuspended cells were stained with 0.016% Trypan Blue and counted. Cell viability was >80%. 0.25 mL of a 1 × 10^6^ viable cells/ml cell suspension was added to 16 mm diameter wells of an uncoated polystyrene plate. The prewarmed plate was placed in a 37 °C incubator with 5% CO_2_ atm with 100 oscillations/min shaking. After 5 min, 0.25 mL of 2 μM test compound in prewarmed HBSS was added per well and mixed to start the reactions with duplicate reactions per compound. Verapamil as the positive control and cell-free reactions were negative controls. At each time point (0, 2, 5, 10, 30, 60, and 120 min), 50 μL aliquots were transferred to a 96-well stop plate containing 150 μL/well of acetonitrile, 0.1% formic acid and 0.1 μM propafenone. The plate was centrifuged for 10 min at 2300 × *g*. 150 μL of the supernatant was transferred to a mass spect sample plate. Samples were analyzed by UPLC/MS as described. Results were expressed as T1/2 (min) and in vitro Clint (μL/min/10^6^ cells). CLint (μL/min/10^6^ cells) estimates were determined using a modified method of McGinnity et al.^37^

Liver microsome membranes stability assay. Assays were conducted in 96-well (1 mL) polypropylene plates. In duplicate, test compounds (1 μM) were incubated in 0.5 mL of 100 mM potassium phosphate buffer (pH 7.4) with 0.5 mg/mL pooled liver microsomes from the test species (Gibco; mouse, CD1, male, MSMCPL; rat, Sprague-Dawley, male, RTMCPL; dog, Beagle, male, DGMCPL; human, mixed gender, HMMCPL), 2 mM tetrasodium NADPH and 3 mM MgCl_2_ for 60 min in a Labnet Vortemp incubator at 37 °C with gentle shaking. At five time points, 75 mL of reaction mixtures were transferred to a 96-shallow-well stop plate on ice containing 225 mL acetonitrile with 0.05 mM propafenone (internal standard). A control reaction (lacking NADPH) was incubated for 60 min at 37 °C to demonstrate NADPH-dependency of compound loss. Verapamil was the positive reference compound (typical T1/2 = 3 min). Standard curves for test compounds were generated using 5 concentrations in duplicate. Stop plates were centrifuged at 2270 × *g* for 10 min and 170 mL of the supernatants were transferred to a Waters Acquity UPLC 700 mL 96-well sample plate, sealed with a cap mat and analyzed by UPLC/MS as described. GraphPad Prism v 5.04 was used for nonlinear fitting of time course data to generate T1/2 (min) and in vitro Clint (μL/min/mg) values.

### Pharmacokinetics

For the pharmacokinetics study, CD-1 male mice (6–8 weeks) were dosed by IV or PO at the indicated concentrations of the indicated compounds dissolved in 5% DMSO, 5% ethanol, 20% TPGS, 30% PEG400, 40% water. Plasma samples were collected at the indicated time points and the indicated compound levels were determined by using standard protocols with a calibration curve and LC/MS. The pharmacokinetic studies was carried out by Pharmaron.

### In Vivo Efficacy Studies

For the MDA-MB-436 in vivo study, 10 × 10^6^ MDA-MB-436 cells were injected with Matrigel subcutaneously into female BALB/c mice 6–8 weeks old. When average tumor size approached the target range of 178 mm^3^—designated as day 1 of the study—tumor size-matched animals were sorted into the treatment groups. Mice were treated with vehicle, 45 mg/kg olaparib (PO,qd) (dissolved in 10% DMSO, 10% solutol, 80% water) with or without 60 mg/kg RTx-303 (PO,bid) (dissolved in 5% DMSO, 5% ethanol, 20% TPGS, 30% PEG400, 40% water) for 56 days. Tumor size and weight measurements were taken twice per week. The cohorts were terminated on day 56 or when tumor volume reached 1500 mm^3^. The mice were observed frequently for overt signs of any adverse, treatment-related side effects, and clinical signs were recorded when observed. Acceptable toxicity was defined as a group mean body weight loss of less than 10% during the study. We did not observe any treatment-related death, nor significant decrease of body weight. In vivo studies were carried out by WuXi AppTec.

For the BR-05-0566 BRCA2 mutant PDX study, ∼30 mm^3^ tumor fragment was implanted subcutaneously into female BALB/c mice 6–8 weeks old. When average tumor size approached the target range of 173 mm^3^—designated as day 1 of the study—tumor size-matched animals were sorted into the treatment groups. Mice were treated with vehicle, 45 mg/kg olaparib (PO,qd) (dissolved as above), 60 mg/kg RTx-303 (PO,bid) (dissolved as above), or 45 mg/kg olaparib with 60 mg/kg RTx-303 (PO,bid) for 28 days. Tumor size and weight measurements were taken twice per week. The six cohorts were terminated on day 28 or when tumor volume reached 1,500 mm^3^. The mice were observed frequently for overt signs of any adverse, treatment-related side effects, and clinical signs were recorded when observed. Acceptable toxicity was defined as a group mean body weight loss of less than 10% during the study. We did not observe any treatment-related death, nor significant decrease of body weight. In vivo studies were carried out by WuXi AppTec.

For the BR-05-0568 BRCA2 mutant PDX study, ∼30 mm^3^ tumor fragment was implanted subcutaneously into female BALB/c mice 6–8 weeks old. When average tumor size approached the target range of 157 mm^3^—designated as day 1 of the study—tumorsize-matched animals were sorted into the three treatment groups. Mice were treated with vehicle, 0.12 mg/kg talazoparib (PO,qd) with and without 60 mg/kg RTx-303 (PO,bid) for 42 days. Tumor size and weight measurements were taken twice per week. The three cohorts were terminated on day 60 or when tumor volume reached 1500 mm^3^. The mice were observed frequently for overt signs of any adverse, treatment-related side effects, and clinical signs were recorded when observed. Acceptable toxicity was defined as a group mean body weight loss of less than 10% during the study. We did not observe any treatment-related death, nor significant decrease of body weight. In vivo studies were carried out by WuXi AppTec.

### Chemical Synthesis

All compounds were >95% pure by HPLC analysis. Synthesis of RTx-283 (Scheme 1). 1-(2-Hydroxy-3,5-bis­(trifluoromethyl)­phenyl)­imidazolidine-2-one (Int-1). To a solution of compound 1 (3.0 g, 8.45 mmol, 1.0 equiv) and compound 2 (1.45 g, 16.9 mmol, 2.0 equiv) in DMA (15 mL) were added CuI (803 mg, 4.23 mmol, 0.5 equiv), DMEDA (744 mg, 8.45 mmol, 1.0 equiv), K2CO3 (2.35 g, 16.9 mmol, 2.0 equiv) and CsF (2.57 g, 16.9 mmol, 2.0 equiv) at rt. The reaction mixture was stirred at 100 °C in an oil-bath for 4 h. After completion of the reaction by TLC (Rf = 0.2, PE/EtOAc = 3/1), water (50 mL) was added and adjust to pH 6 with 1 N HCl. The mixture was extracted with EtOAc (100 mL × 2). The combined organic layer was washed with brine, dried over Na2SO4 and filtered. The filtrate was concentrated under reduced pressure. The residue was purified by column chromatography (100–200 silica gel, 40% EtOAc in PE as eluent) to afford Int-1 as pale green solid (1.0 g, 37%).

**Scheme 1:**
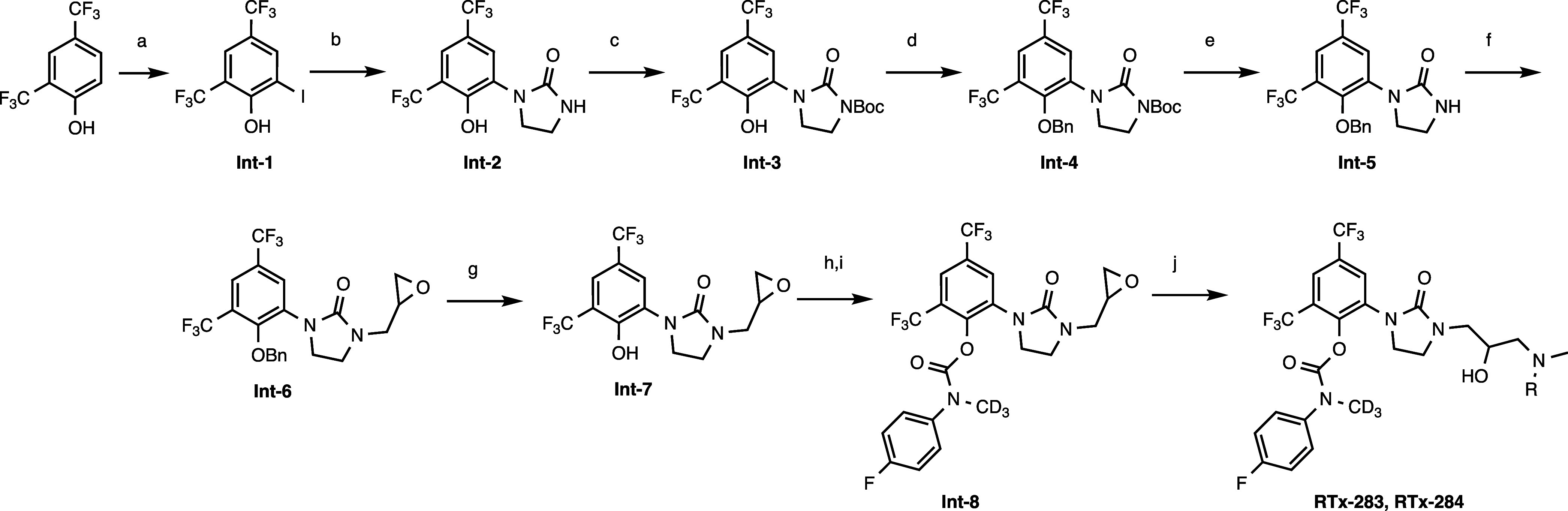
(a) Na_2_CO_3_, I2, THF:H_2_O, 3:1; (b) Imidazolidin-2-one, CuI, DMEDA, K2CO3, CsF, DMA, 100°C, 4 h; (c) Boc2O, TEA, DMAP, DMF, 2 h; (d) BnBr, K2CO3, DCM, 70°C, 3 h; (e) HCl/Dioxane, 1 h; (f) 2-(Bromomethyl)­oxirane, NaH, DMF, 2 h; (g) Pd/C (30%), Dioxane, H2; (h) DIEA, Triphosgene, DCM; (i) 4-Fluoro-*N*-(methyl-d3)-aniline, DCM; (j) MeNH2 or Me_2_NH, THF

Tert-butyl 3-(2-hydroxy-3,5-bis­(trifluoromethyl)­phenyl)-2-oxoimidazolidine-1-carboxylate (Int-2). To a solution of Int-1 (3.0 g, 8.45 mmol, 1.0 equiv) and imidazolidin-2-one (1.45 g, 16.9 mmol, 2.0 equiv) in DMA (15 mL) were added CuI (803 mg, 4.23 mmol, 0.5 equiv), DMEDA (744 mg, 8.45 mmol, 1.0 equiv), K2CO3 (2.35 g, 16.9 mmol, 2.0 equiv) and CsF (2.57 g, 16.9 mmol, 2.0 equiv) at rt. The reaction mixture was stirred at 100 °C in an oil-bath for 4 h. After completion of the reaction by TLC (Rf = 0.2, PE/EtOAc = 3/1), water (50 mL) was added and adjust to pH 6 with 1 N HCl. The mixture was extracted with EtOAc (100 mL × 2). The combined organic layer was washed with brine, dried over Na2SO4 and filtered. The filtration was concentrated under reduced pressure. The residue was purified by column chromatography (100–200 silica gel, 40% EtOAc in PE as eluent) to afford Int-2 as pale green solid (1.0 g, 37%).

Tert-butyl 3-[2-hydroxy-3,5-bis­(trifluoromethyl)­phenyl]-2-oxoimidazolidine-1-carboxylate (Int-3). To a solution of Int-2 (1.0 g, 3.18 mmol, 1.0 equiv) in DMF (10 mL) were added TEA (386 mg, 3.82 mmol, 1.2 equiv), DMAP (40 mg, 0.318 mmol, 0.1 equiv) and Boc2O (728 mg, 3.50 mmol, 1.05 equiv) at rt. The reaction was stirred for 2 h at rt. After completion of the reaction by TLC (Rf = 0.4, PE/EtOAc = 3/1). The mixture was extracted with EtOAc (50 mL × 2). The combined organic layer was washed with brine, dried over Na2SO4 and filtered. The filtrate was concentrated under reduced pressure. The residue was purified by column chromatography (100–200 silica gel, 35% EtOAc in PE as eluent) to afford Int-3 (500 mg, 38%) as off-white solid.

Tert-butyl 3-(2-benzyloxy-3,5-bis­(trifluoromethyl)­phenyl)-2-oxoimidazolidine-1-carboxylate (Int-4). To a solution of Int-3 (566 mg, 1.37 mmol, 1.0 equiv) in DCM (10 mL) were added K2CO3 (286 mg, 2.05 mmol, 1.5 equiv) and BnBr (351 mg, 2.05 mmol, 1.5 equiv) at rt. The reaction was stirred at 70 °C for 3 h. After completion of the reaction by TLC (Rf = 0.9, PE/EtOAc = 3/1), the reaction solution was poured into water (30 mL). The mixture was extracted with EtOAc (50 mL × 2). The combined organic layer was washed with brine, dried over Na2SO4 and filtered. The filtrate was concentrated under reduced pressure. The residue was purified by column chromatography (100–200 silica gel, 5% EtOAc in PE as eluent) to afford Int-4 as pale yellow solid (460 mg, 66%).

1-(2-Benzyloxy-3,5-bis­(trifluoromethyl)­phenyl)-2-oxoimidazolidine (Int-5). Int-4 (460 mg, 0.91 mmol, 1.0 equiv) was added into HCl/dioxane (4 M, 5 mL) at 0 °C. The reaction was stirred for 1 h at rt. After completion of the reaction by TLC (Rf = 0.3, PE/EtOAc = 3/1), the mixture was concentrated to give Int-5 (405 mg, 100%) as off-white solid.

1-(2-(Benzyloxy)-3,5-bis­(trifluoromethyl)­phenyl)-3-(oxiran-2-ylmethyl)­imidazolidine-2-one (Int-6). To a solution of Int-5 (405 mg, 1.0 mmol, 1.0 equiv) in DMF (10 mL) was added NaH (80 mg, 2.0 mmol, 2.0 eq, 60%) at 0 °C. The reaction mixture was stirred for 30 min at rt under N2 atm. 2-(bromomethyl)­oxirane (206 mg, 1.5 mmol, 1.5 equiv) was added to the mixture at 0 °C. The reaction mixture was stirred for 2 h at rt under N2 atm. After completion of the reaction by TLC (Rf = 0.4, PE/EtOAc = 3/1), the mixture was extracted with EtOAc (50 mL × 2). The combined organic layer was washed with brine, dried over Na2SO4 and filtered. The filtrate was concentrated under reduced pressure. The residue was purified by column chromatography (100–200 silica gel, 40% EtOAc in PE as eluent) to afford compound Int-6 (380 mg, 82%) as colorless oil.

1-(2-Hydroxy-3,5-bis­(trifluoromethyl)­phenyl)-3-(oxiran-2-ylmethyl)­imidazolidine-2-one (Int-7). To a solution of Int-6 (380 mg, 0.83 mmol, 1.0 equiv) in dioxane (10 mL), was added Pd/C (114 mg, 30 wt %) at rt under H2 atm. The reaction was stirred for 1 h at rt. After completion of the reaction by TLC (Rf = 0.2, PE/EtOAc = 3/1), the mixture was concentrated under reduced pressure to afford Int-7 as pale-yellow oil (300 mg, 98%).

2-(3-(Oxiran-2-ylmethyl)-2-oxoimidazolidin-1-yl)-4,6-bis­(trifluoromethyl)­phenyl (4-fluorophenyl)­(methyl-d3)­carbamate (Int-8). To a solution of Int-7 (330 mg, 0.89 mmol, 1.0 equiv) in DCM (20 mL), was added DIEA (172 mg, 1.34 mmol, 1.5 equiv) and triphosgene (132 mg, 0.45 mmol, 0.5 equiv) in DCM (5 mL) at −10 °C. The reaction mixture was stirred for 1 h at rt under N2 atm. The reaction solution was concentrated under reduced pressure to remove the solvent. Then DCM (5 mL) was added to above residue. The solution was used for next step. To a solution of 4-fluoro-*N*-(methyl-d3)-aniline (225 mg, 1.34 mmol, 1.5 equiv) in DCM (20 mL) was added the above residue solution at 0 °C. The mixture was stirred for 1 h at rt under N2 atm. After completion of the reaction by TLC (Rf = 0.8, PE/EtOAc = 3/1), HCl (1 M, 15 mL) was added. Then the mixture was extracted with DCM (40 mL × 2). The combined organic layer was washed with brine, dried over Na2SO4 and filtered. The filtrate was concentrated under reduced pressure. The residue was purified with FCC to give Int-8 (240 mg, 51%) as off-yellow oil.

2-(3-(2-Hydroxy-3-(methylamino)­propyl)-2-oxoimidazolidin-1-yl)-4,6-bis­(trifluoromethyl)­phenyl (4-fluorophenyl)­(methyl-d3)­carbamate (RTx-283). To a solution of Int-8 (100 mg, 0.19 mmol, 1.0 equiv) in THF (2 mL) was added MeNH2/THF 2 mL at rt. The reaction was stirred for 24 h at rt. After completion of the reaction by TLC (Rf = 0.4, DCM/MeOH = 10/1), the mixture was concentrated under reduced pressure. The residue was purified by Prep-HPLC to afford RTx-283 (29.8 mg, 28%) as white solid. 1H NMR (400 MHz, CD3OD): δ 8.08 (s, 1H), 7.93–7.86 (m, 1H), 7.40 (s, 2H), 7.17 (t, 2H), 4.10 (br, 1H), 3.73–3.70 (m, 4H), 3.37–3.35 (m, 2H), 3.14–3.00 (m, 2H), 2.71–2.65 (m, 3H); LC-MS: 556.25 (M+H)+.

Synthesis of RTx-284 (Scheme 1). 2-{3-[3-(Dimethylamino)-2-hydroxypropyl]-2-oxoimidazolidin-1-yl}-4,6-bis­(trifluoromethyl)­phenyl *N*-(4-fluorophenyl)-*N*-(methyl-d3)-carbamate (RTx-284). To a solution of compound Int-8 (100 mg, 0.19 mmol, 1.0 equiv) in THF (2 mL) was added Me2NH (2 mL, 2 M in THF) at rt. The reaction was stirred for 30 h at rt. After completion of the reaction by TLC (Rf = 0.4, DCM/MeOH = 10/1), the mixture was concentrated under reduced pressure. The residue was purified by Prep-HPLC to afford RTx-284 (33.4 mg, 30%) as white solid. 1H NMR (400 MHz, CD3OD) δ 8.09–8.08 (m, 1H), 7.92–7.85 (m, 1H), 7.39 (s, 2H), 7.16 (t, 2H), 4.21 (s, 1H), 3.85–3.68 (m, 4H), 3.39–3.37 (m, 2H), 3.19–3.14 (m, 2H), 2.90–2.85 (m, 6H); LC-MS: 570.30 (M+H)+.

Synthesis of RTx-290 (Scheme 2). 2-Iodo-4,6-bis­(trifluoromethyl)­phenyl (4-fluorophenyl)­(methyl)­carbamate (Int-9). To a cold (0 °C) solution of triphosgene (1.6 g, 0.0128 mol) in DCM (40 mL) added a solution of *N*-methyl-4-fluoro aniline (1.89 g, 0.0064 mol) and pyridine (2.52 g, 2.6 mL, 0.032 mol) dropwise for over a period of 10 min. After that continued stirring at RT for 16 h. Progress of the reaction was monitored by TLC (Rf – 0.7, 10% EtOAc in Hexane (×4)). After completion of the reaction, quenched the reaction mixture with 1 M aq. HCl (50 mL) and then extracted with DCM (2 × 50 mL). The DCM layer separated was dried over anhydrous Na2SO4, filtered, and concentrated under reduced pressure to afford (4-fluorophenyl)­(methyl)­carbamic chloride as green solid (1.8 g, 75%). To a stirred solution of Int-1, (0.5 g, 0.0014 mol) in pyridine (10 mL) at RT added (4-fluorophenyl)­(methyl)­carbamic chloride (0.34 g, 0.0018 mol) and continued stirring at 80 °C for 4 h. After completion of the reaction was confirmed by TLC (Rf – 0.8, 20% EtOAc in Hexane) quenched the reaction mixture with 1 M HCl (50 mL) and extracted with EtOAc (2 × 50 mL). The organic layer separated was combined, dried over anhydrous Na2SO4, filtered, and concentrated under reduced pressure. The crude obtained was purified by silica gel chromatography (100–200 and 5–10% EtOAc in Hexane as eluent) to afford Int-9 as off-white solid (0.66 g, 93%).

**Scheme 2:**
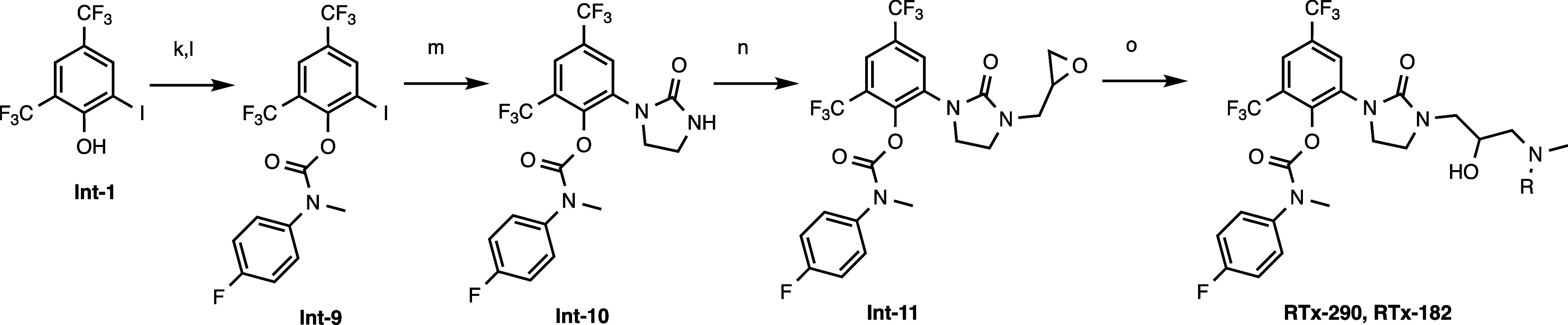
(k) DIEA, Triphosgene, DCM; (l) 4-Fluoro-*N*-methyl-aniline, Pyridine; (m) Imidazolidin-2-one, CuI, DMEDA, K2CO3, CsF, DMA, 90°C, 24 h; (n) K3PO4, epibromohydrin, KI, DMF; (o) MeNH_2_ or Me_2_NH, MeOH

2-(2-Oxoimidazolidin-1-yl)-4,6-bis­(trifluoromethyl)­phenyl (4-fluorophenyl)­(methyl) carbamate (Int-10). Int-9 (0.2 g, 0.00039 mol), 2-imidazilidinone (0.064 g, 0.0007 mol), copper­(I)-iodide (0.037 g, 0.00019 mol), *N*,*N*’-dimethylethylenediamine (0.07 mL, 0.00039 mol), cesium fluoride (0.12 g, 0.00079 mol) and potassium carbonate (0.1 g, 0.00072 mol) were suspended in 1,4-dioxane priorly purged with N2 for 30 min (16 mL). The reaction mixture was heated at 90 °C in an oil-bath for 24 h. After completion of the reaction by TLC (Rf 0.4, 5% MeOH in DCM) the reaction mixture was concentrated under reduced pressure. The residue obtained was purified by column chromatography (100–200 silica gel, 0–3% MeOH in DCM as eluent) to afford Int-10 as off-white solid (50 mg). The solid was repurified by preparative TLC (solid phase: Merck, 20 × 20 cm, silica gel 60 GF254, 1 mm, PLC glass plate, 2% MeOH in DCM as eluent) to afford white solid (28 mg, 15%).

2-(3-(Oxiran-2-ylmethyl)-2-oxoimidazolidin-1-yl)-4,6-bis­(trifluoromethyl)­phenyl (4-fluorophenyl)­(methyl)­carbamate (Int-11). A stirred solution of Int-10 (0.1 g, 0.0002 mol) in DMF (8 mL) was purged with N2 for 10 min. To the above solution added K3PO4 (0.27 g, 0.00128 mol) at RT and continued purging for 10 min. After 30 min of stirring at RT, reaction mixture was cooled to 0 °C and to the same added epibromohydrin (0.12 mL, 0.00128 mol) and KI (0.13 g, 0.0008 mol). The reaction mixture was stirred at RT for 20 h. After completion of the reaction confirmed by TLC (Rf 0.3, 60% EtOAc in Hexane) the reaction mixture was diluted with cold water (150 mL) followed by extraction with ethyl acetate (2 × 100 mL). The organic fractions were combined, dried over anhydrous Na2SO4, filtered and concentrated under reduced pressure. The crude residue was purified by preparative TLC (solid phase: Merck, 20 × 20 cm, silica gel 60 GF254, 1 mm, PLC glass plate, 50% EtOAc in Hexane as eluent) to afford Int-11 as sticky oil (40 mg, 38%).

2-(3-(2-Hydroxy-3-(methylamino)­propyl)-2-oxoimidazolidin-1-yl)-4,6-bis­(trifluoromethyl)­phenyl (4-fluorophenyl)­(methyl)­carbamate (RTx-290). To a solution of Int-11 (0.025 g, 0.047 mmol) in 3 mL anhydrous methanol at 0 °C, 2 M Methylamine (2 mL, 4 mmol) was added. The reaction was stirred for 20 h at room temperature. The solvent was removed under reduced pressure. The oil was dissolved in dichloromethane and triturated with hexanes. The solvent was removed under reduced pressure to afford RTx-290 as semi-solid (26 mg, 100% yield). 1H NMR (400 MHz, MeOD) δ 8.09 (bs, 1H), 7.8 (m, 1H), 7.42 (bs, 2H), 7.17 (m, 2H), 3.98 (bs, 1H), 3.71 (m, 3H), 3.51 (bs, 1H), 3.34 (m, 4H), 3.36 (m, 2H), 2.66 (m, 2H), 2.43 (m, 3H); ESIMS: *m*/*z* 553.24 ((M+H)+).

Synthesis of RTx-182 (Scheme 2). 2-{3-[3-(Dimethylamino)-2-hydroxypropyl]-2-oxoimidazolidin-1-yl}-4,6-bis­(trifluoromethyl)­phenyl *N*-(4-fluorophenyl)-*N*-methylcarbamate (RTx-182). To a stirred solution of Int-11 (0.1 g, 0.00019 mol) in methanol (5 mL) under N2 atm was added a solution of dimethylamine (2 M in THF, 4.75 mL, 0.0095 mol) at 0 °C and then stirring was continued at rt for 6 h. After completion of the reaction confirmed by TLC (Rf 0.2, 7% MeOH in DCM), the reaction mixture was directly concentrated under reduced pressure at 40 °C. The impure residue was purified by preparative TLC (solid phase: Merck, 20 × 20 cm, silica gel 60 GF254, 1 mm, PLC glass plate, 5% MeOH in DCM as eluent) to afford RTx-182 as sticky off-white solid (40 mg, 37%). 1H NMR (400 MHz, DMSO-d6) δ 8.2 (m, 1H), 7.93 (d, J = 21.6 Hz, 1H), 7.44 (s, 2H), 7.28 (t, J = 8.5 Hz, 2H), 4.7 (d, J = 9.9 Hz, 1H), 3.82 (d, J = 22.1 Hz, 2H), 3.46 (m, 3H), 3.42 (s, 2H), 3.07 (s, 1H), 2.28 (s, 2H), 2.20 (s, 6H), 1.29 (m, 1H); MS­(ESI): *m*/*z* 567.35 (M+H)+.

Synthesis of RTx-302 (Scheme 3). 1-[2-(Benzyloxy)-3,5-bis­(trifluoromethyl)­phenyl]-3-{[(2R)-oxiran-2-yl]­methyl}­imidazolidin-2-one (Int-12). To a solution of Int-5 (520 mg, 1.28 mmol, 1.0 equiv) in DMF (5 mL) was added 60% NaH (128 mg, 3.20 mmol, 2.5 equiv) at 0 °C. The reaction mixture was stirred at rt for 30 min. (2R)-2-(chloromethyl)­oxirane (177 mg, 1.92 mmol, 1.5 equiv) was added. The reaction mixture was stirred at rt for 1.5 h. After completion of the reaction by TLC, the reaction solution was poured into water (100 mL). The mixture was extracted with ethyl acetate (150 mL × 2). The combined organic layer was washed with brine, dried over Na2SO4 and filtered. The filtrate was concentrated under reduced pressure. The residue was purified by column chromatography (100–200 silica gel, 45% EA in PE as eluent) to afford Int-12 (500 mg, 84%) as yellow oil.

**Scheme 3:**
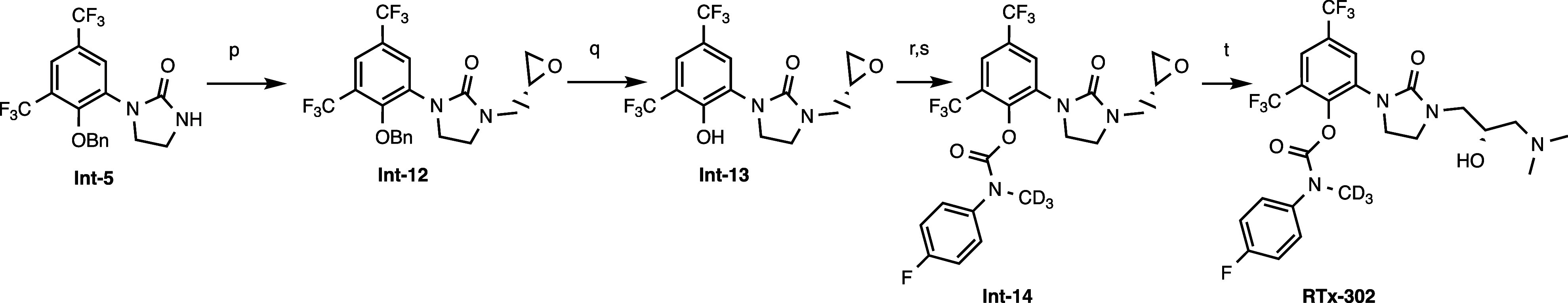
(2R)-2-(Chloromethyl)­oxirane, NaH, DMF (q) Pd/C (20%), Dioxane, H2; (r) DIEA, Triphosgene, DCM; (s) 4-Fluoro-*N*-(methyl-d3)-aniline, DCM; (t) Me_2_NH, THF

1-[2-Hydroxy-3,5-bis­(trifluoromethyl)­phenyl]-3-{[(2R)-oxiran-2-yl]­methyl}­imidazolidin-2-one (Int-13). To a solution of Int-12 (500 mg, 1.08 mmol, 1.0 equiv) in dioxane (5 mL) was added Pd/C (100 mg, 20 wt %) at rt. The reaction mixture was stirred for 1 h at rt. After completion of the reaction by TLC, the mixture was filtered with Celite. The filtrate was concentrated under reduced pressure to afford Int-13 (420 mg, 100%) as colorless oil.

2-(3-{[(2R)-Oxiran-2-yl]­methyl}-2-oxoimidazolidin-1-yl)-4,6-bis­(trifluoromethyl)­phenyl *N*-(4-fluorophenyl)-*N*-(methyl-d3)-carbamate (Int-14). To a solution of Int-13 (420 mg, 1.13 mmol, 1.0 equiv) in DCM (20 mL) were added DIEA (146 mg, 1.13 mmol, 1.0 equiv), triphosgene (335 mg, 1.13 mmol, 1.0 equiv) at −10 °C. The reaction mixture was stirred at rt for 1 h. The reaction solution was concentrated under reduced pressure to remove the solvent. Then DCM (10 mL) was added to above residue. The solution was used for next step. To a solution of compound 10 (218 mg, 1.70 mmol, 1.5 equiv) in DCM (10 mL) was added the above residue solution at 0 °C. The mixture was stirred at rt for 1 h. After completion of the reaction by TLC, the reaction solution was poured into water (100 mL). The mixture was extracted with DCM (150 mL × 2). The combined organic layer was washed with brine, dried over Na2SO4 and filtered. The filtrate was concentrated under reduced pressure. The residue was purified by column chromatography (100–200 silica gel, 65% EA in PE as eluent) to afford Int-14 (400 mg, 67%) as yellow oil.

2-{3-[(2S)-3-(Dimethylamino)-2-hydroxypropyl]-2-oxoimidazolidin-1-yl}-4,6-bis­(trifluoromethyl)­phenyl *N*-(4-fluorophenyl)-*N*-(methyl-d3)-carbamate (RTx-302). To a solution of Int-14 (150 mg, 0.28 mmol, 1.0 equiv) in THF (2 mL) at rt were added Me2NH (0.85 mL1.0 M/L in THF, 3.0 equiv). The mixture was stirred for 16 h at rt. After completion of the reaction by TLC, the mixture was concentrated and the residue was purified by column chromatography (100–200 silica gel, 9.5% MeOH in DCM as eluent) to afford RTx-302 (25.0 mg, 15%) as off-white solid. 1H NMR (400 MHz, CD3OD) δ 8.08 (br, 1H), 7.83 – 7.90 (m, 1H), 7.40 (s, 2H), 7.15 (t, J = 8.4 Hz, 2H), 3.98–3.71 (m, 5H), 3.38–3.33 (m, 1H), 3.27–3.21 (m, 1H), 2.49 (br s, 2H), 2.37 – 2.33 (m, 6H); LC-MS: 570.1 (M+H)+.

Synthesis of RTx-303 (Scheme 4): RTx-284 (15.5 g, 27.22 mmol, 1 equiv) was separated by Chiral SFC (column: DAICEL CHIRALPAK IG (250 mm × 50 mm,10 μm); mobile phase: [CO2-EtOH­(0.1%NH4OH)];B%:10%, isocratic elution mode) and further by Chiral SFC (column: DAICEL CHIRALPAK IG (250 mm × 50 mm,10 μm); mobile phase: [CO2-EtOH­(0.1%NH3H2O)];B%:10%, isocratic elution mode) to afford desired product RTx-303 (6.50 g, 11.39 mmol, 83.69% yield) as an off-white solid and another desired product RTx-302 (5.5 g, 9.63 mmol, 70.78% yield, 99.73% purity) as an off-white solid.

**Scheme 4:**
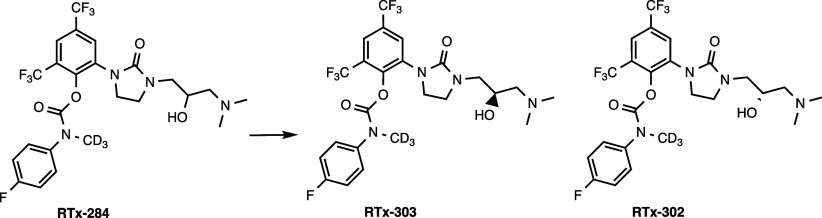
Purification of Enantiomers by Chiral Supercritical Fluid Chromatography (SFC)

LCMS RTx-303–4.5G: RT = 0.842 min, *m*/*z* 570.2 [M + H]^+^

HPLC RTx-303–4.5G: RT = 2.431 min, 99.79% purity

^1^H NMR RTx-303 (400 MHz, DMSO-*d*_6_) δ 8.10 (1H, m), 7.85–7.94 (1H, m), 7.44 (2H, s), 7.15–7.19 (2H, t, *J* = 8.4 Hz), 3.52–4.08 (5H, m), 3.36–3.41 (1H, m), 3.20–3.31 (1H, m), 2.41 (2H, s), 2.32 (6H, s).

## Supplementary Material





## Data Availability

All data needed to evaluate the conclusions in the paper are present in the paper and/or the Supporting Information. Additional data related to this paper can be requested from the authors.

## Notes

Molecular formula strings for all compounds reported (CSV)
X-ray crystallography data collection, refinement and validation statistics; clonogenic survival data for RTx-303; synergy plots for RTx-303 niraparib combinations; analyses of X-ray structures of Polθ-pol bound to DNA and RTx-161 and RTx-303; RTx-303 pharmacological profiling data against kinase panel; in vitro DNA polymerase selectivity data for RTx-284 and RTx-303; PK data for RTx-303, RTx-302 and RTx-284; solubility and stability data for RTx-284 and RTx-283 (PDF)

## Abbreviations

BRCA: BRCA1/2
DSBs: double-strand breaks
HR: homologous recombination
HRD: HR-deficient
MMEJ: microhomology-mediated DNA end-joining
PARP1: Poly-ADP ribose polymerase 1
PARPi: PARP inhibitors
Polq-hel: Polq helicase
Polq: DNA polymerase q
Polqi: Polq inhibitors
Polq-pol: Polq polymerase
ssDNA: single-strand DNA
SL: synthetic lethality
T1/2: half-life
TMEJ: theta-mediated end-joining
