# Supplementary material for: RTx-303, an Orally Bioavailable Polθ Polymerase Inhibitor That Potentiates PARP Inhibitors in BRCA Mutant Tumors
Source: J Med Chem. 2025 Oct 22;68(21):22196–215. doi: 10.1021/acs.jmedchem.5c00551 (PMC12621202; doi:10.1021/acs.jmedchem.5c00551)
Supplement: Supplementary file 2 [file jm5c00551_si_016.pdf]

Supporting Information

RTx-303, an Orally Bioavailable Polθ Polymerase Inhibitor that Potentiates PARP Inhibitors in BRCA-Mutant Tumors

Gurushankar Chandramouly[1,3], William Fried[1,2], John Gordon[6], Douglas Ralph[3], Chanitta Keuk[6], Sangeeta Kumari[6], Mercy Ramanjulu[4], William Auerbacher[3], Leonid Minakhin[3], Taylor Tredinnick[3], Bernadette Tiberi[3], George Morton[7], Robert Betsch[5], Kathy Q. Cai[5], Umeshkumar M. Vekariya[6], Mrityunjay Tyagi[3], Tomasz Skorski[5,6], Sergey Karakashev[6], Neil Johnson[5], Wayne E. Childers Jr.[4,7], Xiaojiang S. Chen[2,4], and Richard T. Pomerantz[3,4*]

Affiliations:

[2]Molecular and Computational Biology, USC Dornsife Department of Biological Sciences, University of Southern California, Los Angeles, CA, 90089, USA

[3]Department of Biochemistry and Molecular Biology, Sidney Kimmel Cancer Center, Thomas Jefferson University, Philadelphia, PA 19107, USA

[4]Recombination Therapeutics, Pennsylvania Biotechnology Center, Doylestown, PA 18902, USA

[5]Nuclear Dynamics and Cancer Program, Fox Chase Cancer Center, Philadelphia, PA 19111, USA

[6]Fels Cancer Institute for Personalized Medicine, Temple University Lewis Katz School of Medicine, Philadelphia, PA, 19140, USA

[7]Temple University, School of Pharmacy, Philadelphia, PA, 19140, USA

*Correspondence to: Richard T. Pomerantz, richard.pomerantz@jefferson.edu

[1]Authors contributed equally to manuscript.

Contents of supplementary information:

1. X-ray crystallography data collection, refinement, and validation statistics
2. Solubility and stability data for RTx-284 and RTx-283
3. Comparison of X-ray structures of Polθ-pol bound to RTx-161 and RTx-302
4. DNA Polymerase selectivity data for RTx-284
5. Pharmacodynamics analysis of RTx-284 and olaparib
6. PK data for RTx-302 and RTx-303, and their respective metabolites
7. DNA Polymerase selectivity data for RTx-303
8. Pharmacological profiling data for RTx-303 against kinases
9. Clonogenic survival data for RTx-303 and other Polθ-pol inhibitors
10. IC_{50} data for RTx-303 in HR-proficient and HRD cells
11. Synergy plots for RTx-303 and niraparib in HRD cells
12. Mouse body weight data for RTx-303 treatments
13. LC-MS and NMR data for all compounds

	Polθ-pol:DNA:RTX-161	Polθ-pol:DNA:RTX-302
**Data Collection and processing**		
Space group	P 31 2 1	P 32 2 1
**Cell dimensions**		
a,b,c (Å)	172.356 172.356 62.4148	171.119 171.119 118.026
α, β, γ (°)	90, 90, 120	90 90 120
Resolution (Å)	74.63 - 3.31 (3.429-3.31)	74.1 - 2.43 (2.517-2.43)
R_{merge}	0.2182 (2.092)	0.2405 (3.954)
CC1/2	0.999 (0.656)	0.998 (0.37)
I/sigma(I)	8.27 (0.49)	12.02 (0.62)
Completeness (%)	99.21 (100.00)	99.98 (99.97)
Total observations	320437 (32786)	1528141 (153112)
Unique observations	16005 (1592)	75126 (7434)
Redundancy	20.0 (20.6)	20.3 (20.6)
**Refinement Statistics**		
Resolution (Å)	74.63 - 3.31	74.1 - 2.43
R_{work}/R_{free} (%)	21.02/24.16	20.97/23.73
No. atoms	5489	12006
B factors ($Å^2$)	142.65	72.97
**r.m.s deviations**		
Bond lengths (Å)	0.002	0.03
Bond angles (°)	0.499	0.522

Supplementary Table 1. X-ray crystallography data collection, refinement, and validation statistics

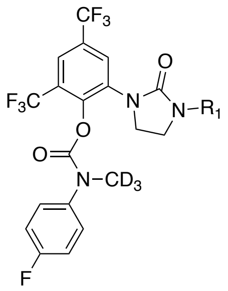

	R1	Solubility (PBS, 2% DMSO)	$T_{1/2}$ PBS	$T_{1/2}$ mouse plasma	$T_{1/2}$ simulated intestinal fluid	$T_{1/2}$ simulated gastric fluid
**RTx-284**	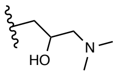	>200 μM	>3 hr	>3 hr	>3 hr	> 3 hr
**RTx-283**	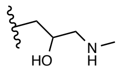	>200 μM	>3 hr	>3 hr	>3 hr	> 3 hr

Supplementary Fig. 2 Solubility and stability of RTx-284 and RTx-283.

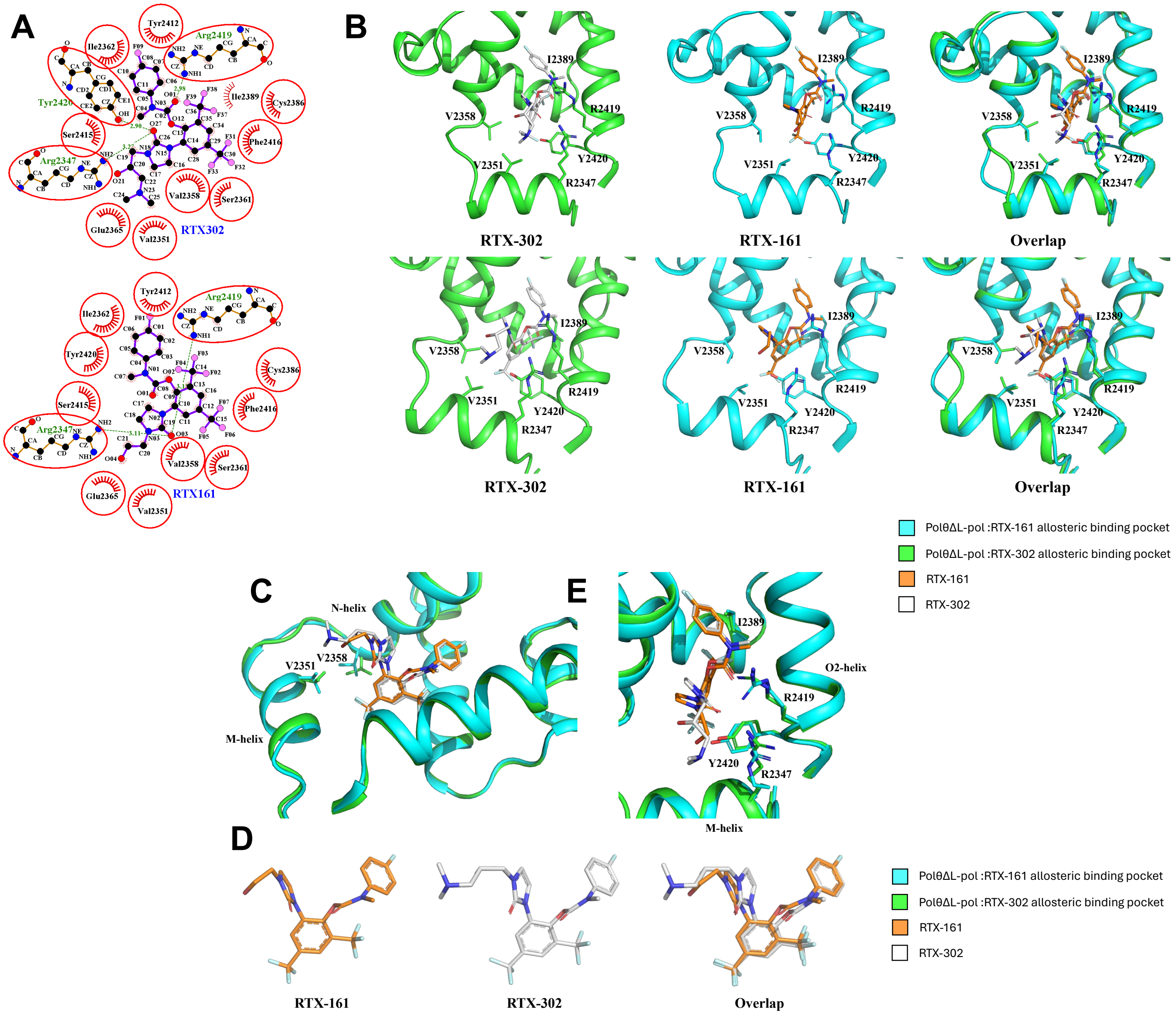

Supplementary Fig. 3 Comparison of RTx-161 and RTx-302 binding to Polθ-pol.

A. 2D ligand interaction plot comparing the interactions between inhibitor RTx-302 and its surrounding allosteric pocket (top) with inhibitor RTx-161 and its surrounding allosteric pocket (bottom) of Polθ-pol. Residues that have contacts or bonds in both plots are circled. **B.** 3D structures of the allosteric binding pocket of Polθ-pol in complex with RTx-302 (green) and RTx-161 (blue). Major differences in interacting residues between the two structures identified from the 2D ligand interaction map are shown as well as residues with significantly different orientations. **C.** While the solvent exposed moiety of RTx-161 pulls body of the inhibitor towards the M-helix of the allosteric binding pocket, the longer side-chain of RTx-302 is able to reach the M-helix and instead pushes the inhibitor slightly away from the M-helix. **D.** 3D confirmations of RTx-161 (orange) and RTx-302 (white) in their respective co-crystal complex structures with Polθ-pol:DNA. **E.** The difference in orientation of RTx-161 and RTx-302 while in the allosteric binding pocket changes the hydrogen bonds that are formed between the inhibitors and the neighboring residues.

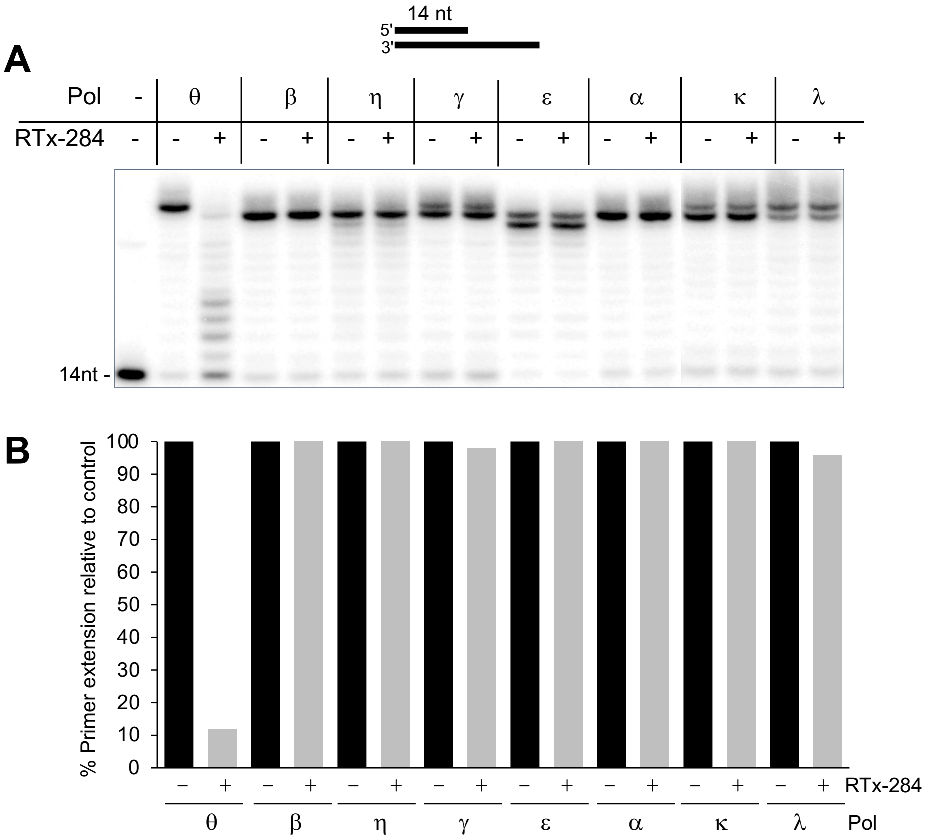

Supplementary Fig. 4 RTx-284 exhibits specific inhibition of Polθ-pol
A. Denaturing gel showing primer extension by the indicated DNA polymerases in the presence or absence of RTx-284. **B.** Bar plot showing % relative extension by the indicated DNA polymerases in the presence and absence of RTx-284.

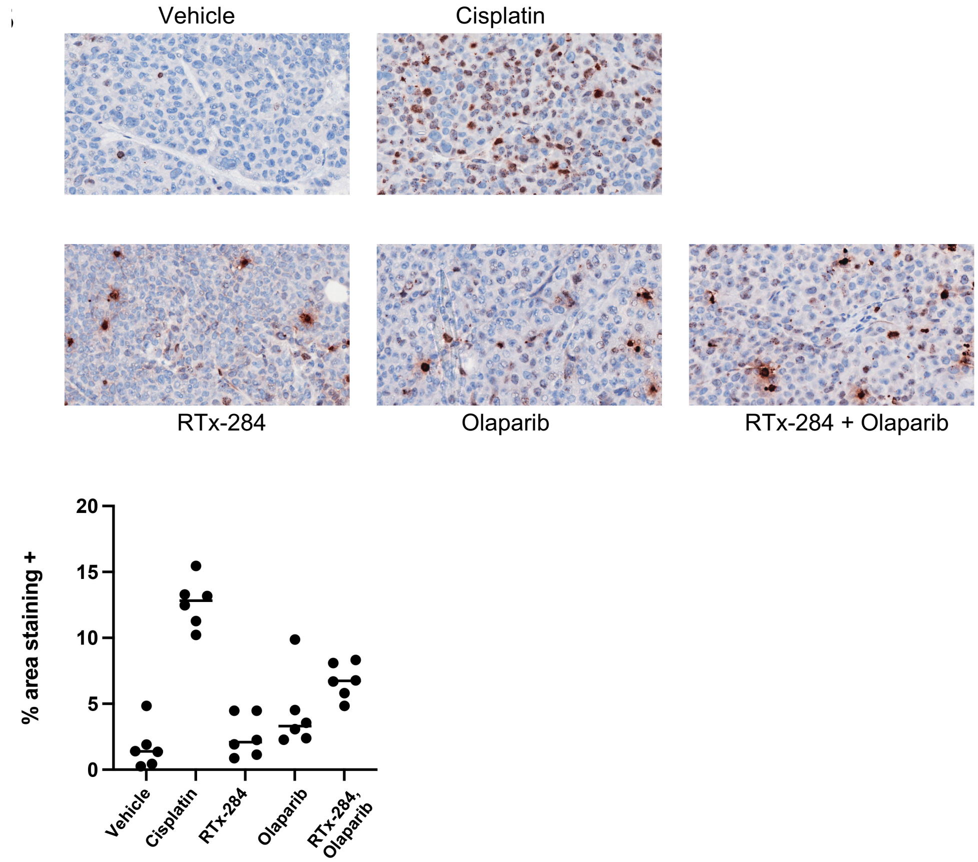

Supplementary Fig. 5 Pharmacodynamics analysis of RTx-284 treatment with and without olaparib in NSG mice. Representative γ-H2AX immunofluorescence images of HCT116 *BRCA2-/-* tumor FFPE samples following treatment with vehicle, 6 mg/kg cisplatin for 2 days, 80 mg/kg (PO,BID) RTx-284 for 3 days, 50 mg/kg (PO,qd) olaparib (50 mg/kg) for 3 days (top). Dot plot showing % area staining of g-H2AX for the indicated treated cohorts (3 mice per cohort)(bottom). Image j was used to calculate the percentage area of an image that stained positive using the same threshold for all images.

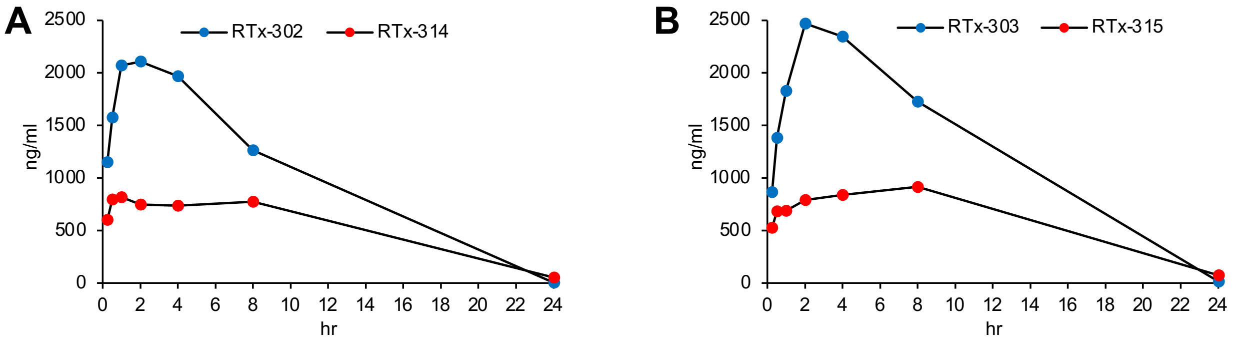

Supplementary Fig. 6 Pharmacokinetics of RTx-302, RTx-303 and their respective metabolites.
A. Scatter plot showing *in vivo* exposure of RTx-302 and metabolite RTx-314 in CD-1 mice following po administration of 50 mg/kg RTx-302. Data represent mean (n = 3). **B.** Scatter plot showing *in vivo* exposure of RTx-303 and metabolite RTx-315 in CD-1 mice following po administration of 50 mg/kg RTx-302. Data represent mean (n = 3).

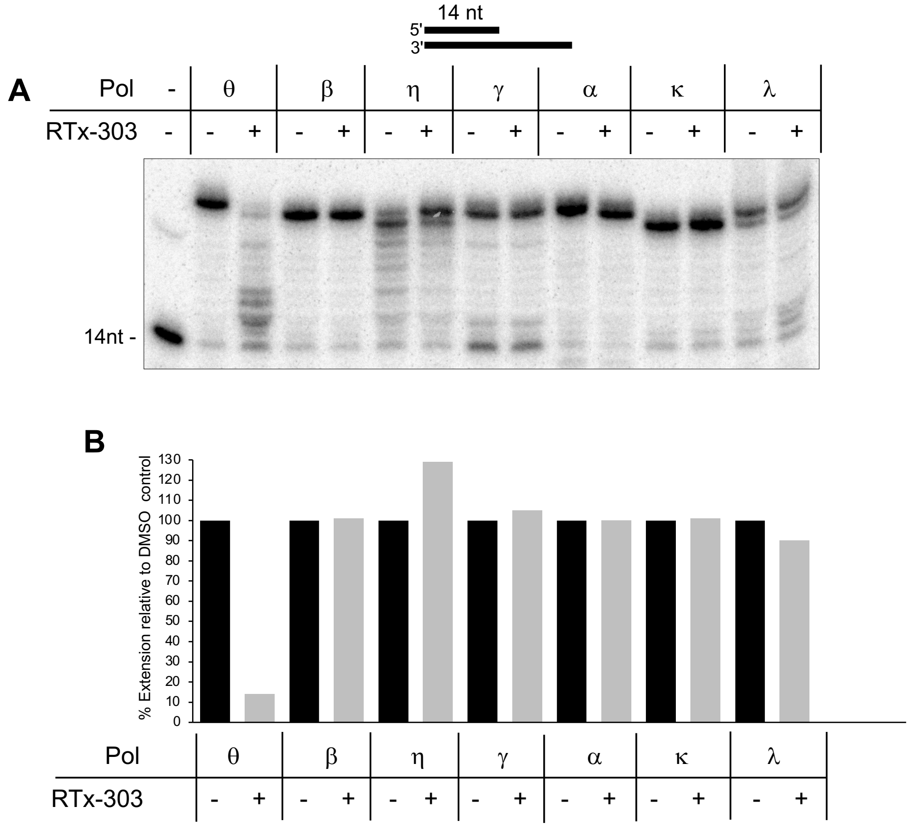

Supplementary Fig. 7 RTx-303 exhibits specific inhibition of Polθ-pol
A. Denaturing gel showing primer extension by the indicated DNA polymerases in the presence or absence of RTx-303. **B.** Bar plot showing % relative extension by the indicated DNA polymerases in the presence and absence of RTx-303.

Kinase	% Activity with 10 μM RTx-303
A-Raf(h)	105
Cdc7/cyclinB1(h)	92
CDK1/cyclinB(h)	105
CDK2/cyclinA(h)	100
CDK2/cyclinE(h)	109
CDK3/cyclinE(h)	114
CDK5/p25(h)	104
CDK6/cyclinD3(h)	101
CDK7/cyclinH/MAT1(h)	109
CDK9/cyclin T1(h)	119
CDK14/cyclinY(h)	105
CDK16/cyclinY(h)	102
CDK17/cyclinY(h)	110
CDK18/cyclinY(h)	89
CDKL4(h)	99
ChaK1(h)	102
DYRK3(h)	113
eEF-2K(h)	94
GRK5(h)	103
GRK6(h)	90
MAPKAP-K3(h)	98
MLK4(h)	109
MOK(h)	100
PASK(h)	75
PEK(h)	104
PDHK2(h)	98
PDHK4(h)	93
PKCα(h)	103
PKCβI(h)	103
PKCγ(h)	111
PKCζ(h)	102
TAF1L(h)	95
TRB2(h)	98
TSSK2(h)	102
TSSK4(h)	117
TTBK1(h)	101
TTBK2(h)	99
VRK1(h)	92
ATM(h)	93
ATR/ATRIP(h)	77
DNA-PK(h)	94
PI3KC2a(h)	79
PIP4K2a(h)	96

Supplementary Fig. 8 Pharmacological profiling of RTx-303 against kinases.
% activity of the indicated kinases is shown following incubation with 10 μM RTx-303 in vitro. Data represent mean (n = 2).

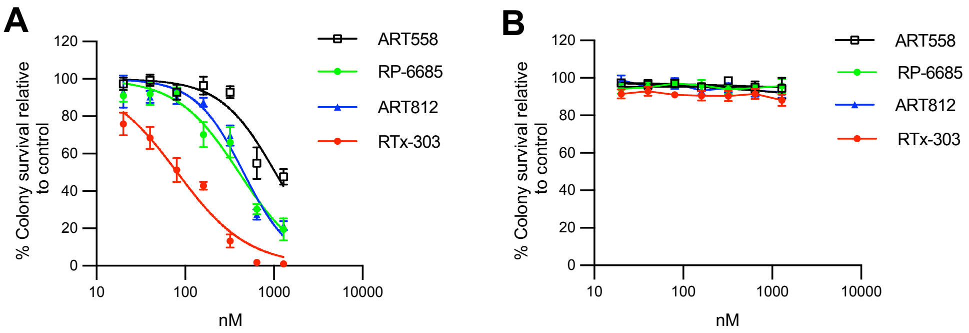

Supplementary Fig. 9 RTx-303 exhibits higher cellular potency than previously developed Polθ-pol inhibitors. Scatter plots showing % colony survival of HCT116 *BRCA2-/-* (**A**) and HCT116 *BRCA2+/+* (**B**) cells following treatment with the indicated concentrations of the indicated inhibitors. Data represent mean of two separate experiments performed in triplicate.

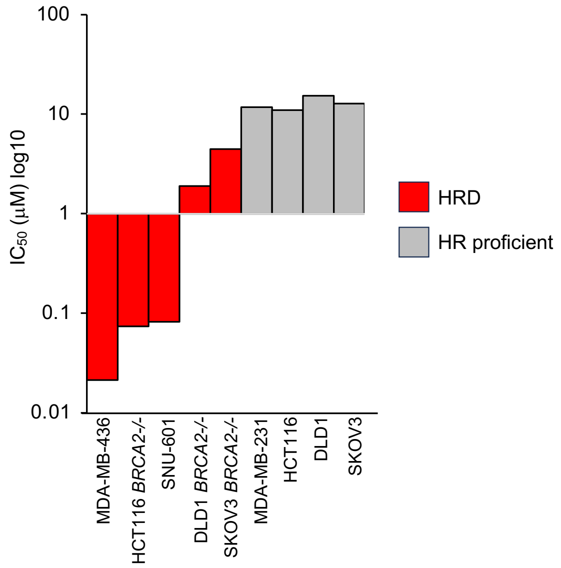

Supplementary Fig. 10 RTx-303 preferentially kills HRD cells.
Bar plot showing IC_{50} of RTx-303 in the indicated HRD (red) and HR proficient (grey) cell lines.

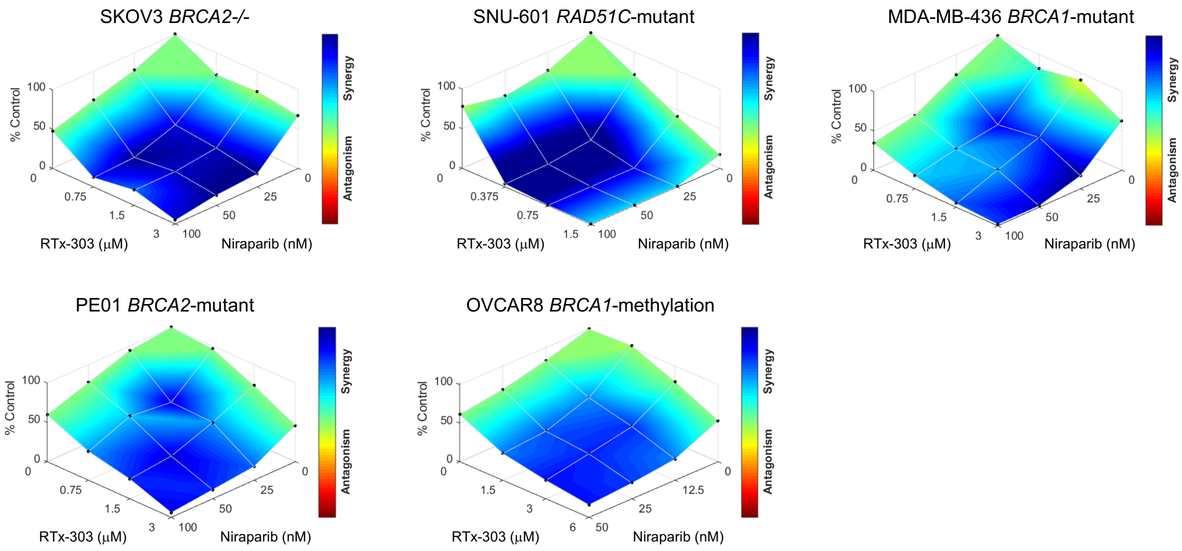

Supplementary Fig. 11 RTx-303 exhibits synergistic activity with niraparib in BRCA-mutant cells.
Synergy plots created by Combenefit software showing synergistic activity between the indicated concentrations of RTx-303 and Niraparib in the indicated HRD cell lines. Data represent mean of at least two separate experiments performed in triplicate.

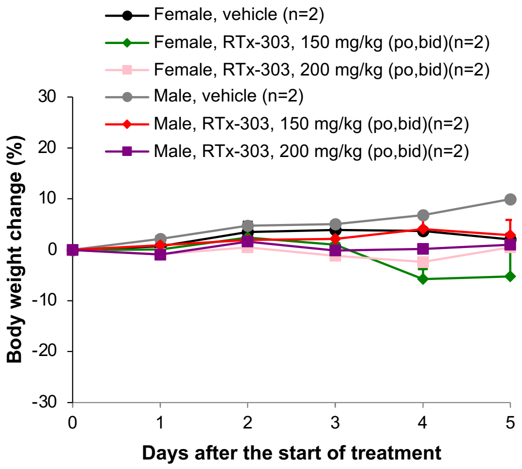

Supplementary Fig. 12 Effect of RTx-303 on mouse body weight.
Scatter plot showing % body weight change following administration of the indicated concentrations of RTx-303 in male and female BALB/c mice. Data represent mean (n=2).

LC-MS and NMR data for RTx-182

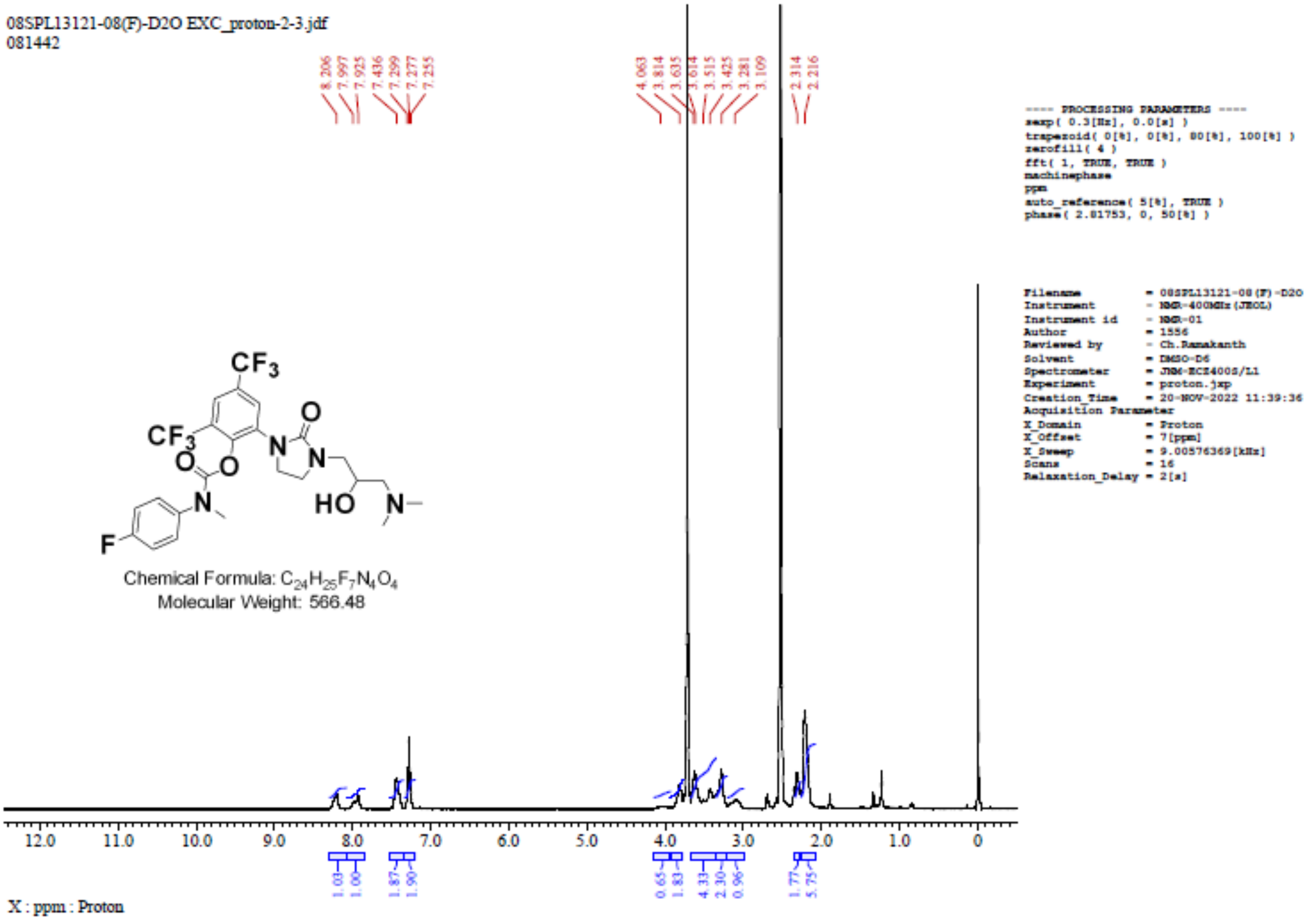

Sample Name : 08SPL13121-08(F)
A.R Number : 081449
Data Filename : 08SPL13121-08(F).lcd
Methoed Filename:Scan Method-General-5-LC.lcm
Batch Filename :LC 19-Nov-2022.lcb
Vial# :20
Injection volume :5 µL Instrument Id : LCMS-MS-01
Data Acquired : 11/20/2022 7:28:04 AM Acquired by : D. Devender
Data Processed :11/20/2022 8:03:07 AM
Method Filename :Scan Method-General-5-LC.lcm

Method Conditions:
Column ID : HPLC-330
Column : Hypersil BDS C18,150x4.6,5µm
Mobile Phase-A : 5mM Ammonium Acetate in Water
Mobile Phase-B : Acetonitrile
Gradient Time : 0.01 10.0 30.0
A% : 95 10.0 10.0
Inj volume : 5µl
Flow Rate : 1.0mL/min
Diluent : Acetonitrile : Methanol (1:1)
Sample Preparation : About 20mg in 20mL of diluent

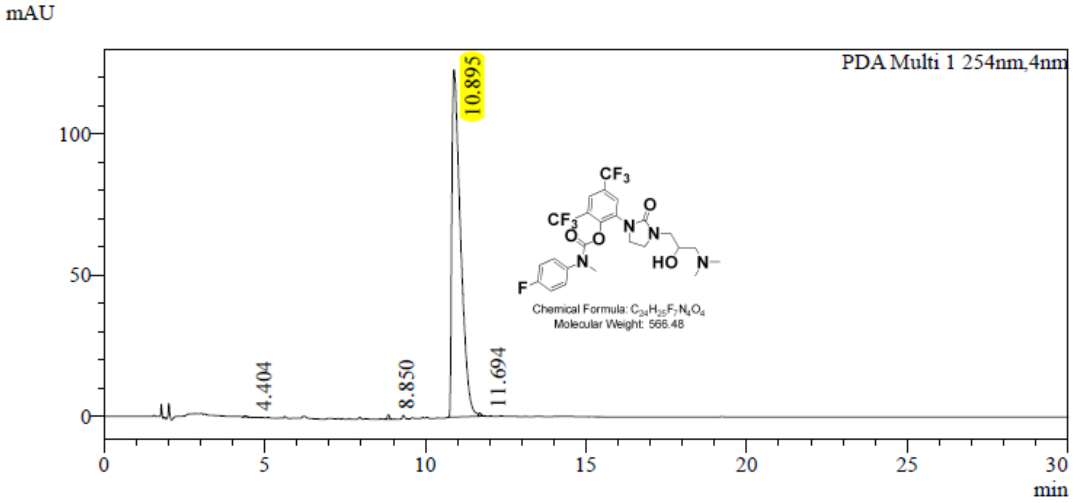

PDA Ch1 254nm

Peak#	Ret. Time	Relative Retention Time	Area	Area%
1	4.40	0.404	1621	0.070
2	8.85	0.812	7003	0.301
3	10.90	1.000	2308126	99.376
4	11.69	1.073	5880	0.253
Total			2322630	100.000

LC-MS and NMR data for RTx-284

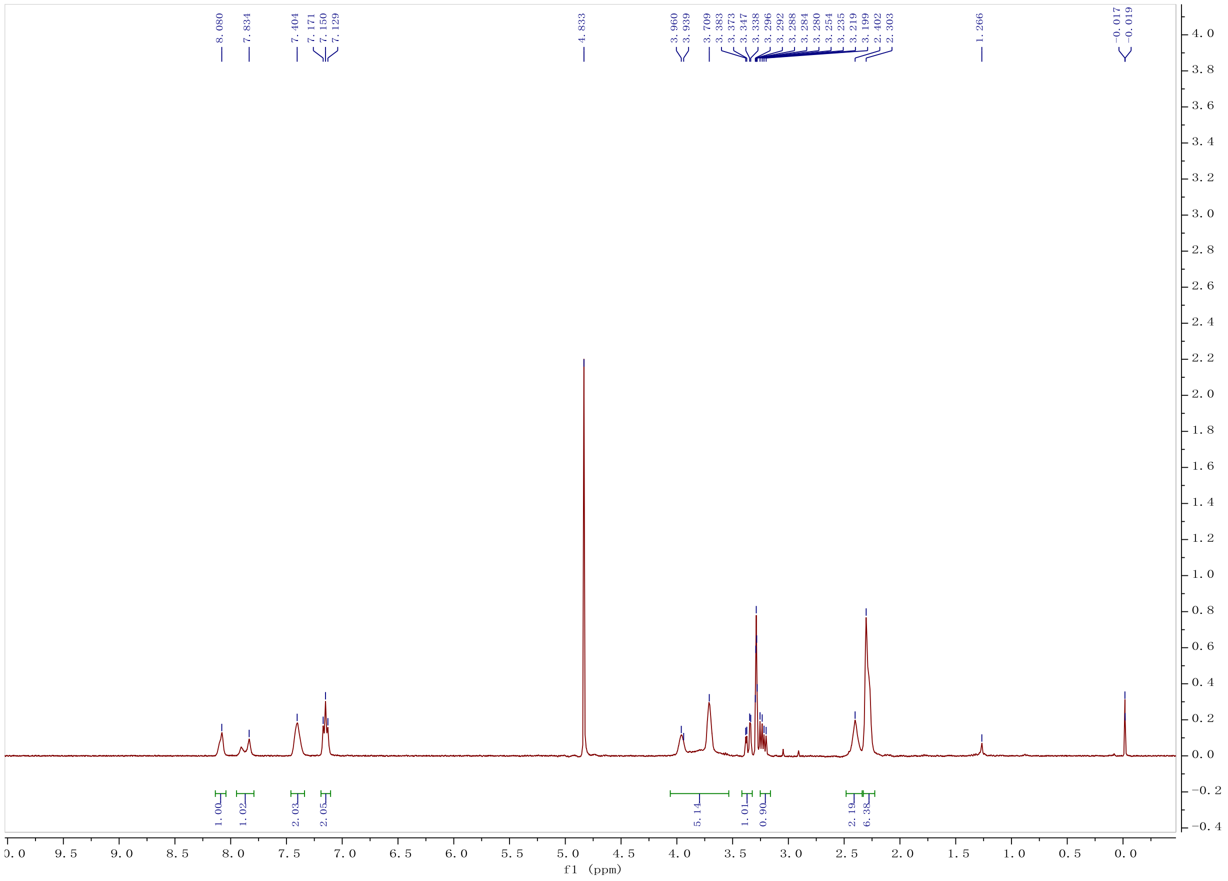

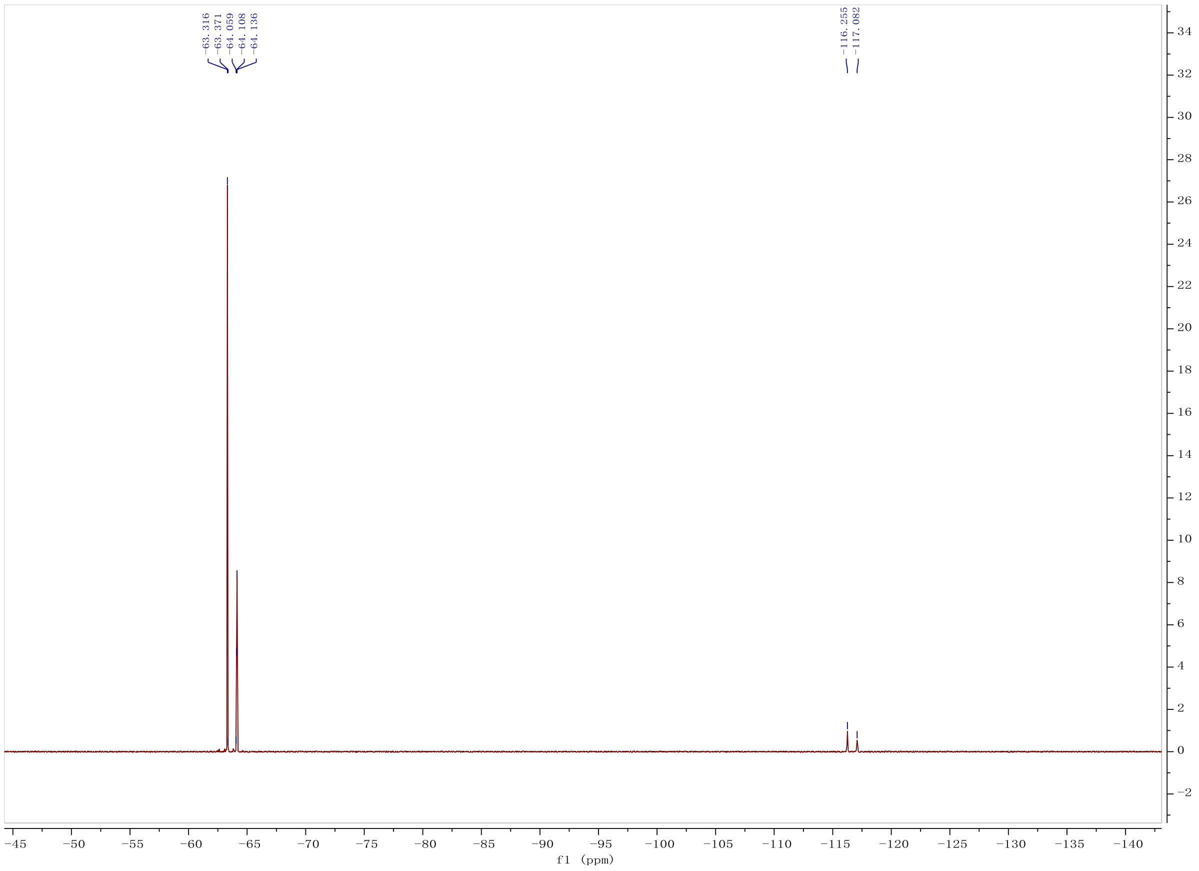
-63.316
-63.371
-64.059
-64.108
-64.136
-116.255
-117.082
f1 (ppm)

LC-MS Report

Sample Name : 3659179
Vial# : 18
Injection Volume : 7
Data File : Z:\data\Data\LCMS007\202308\230802\3659179_1016_005.lcd
Method File : D:\7#lcms\method-2\normal-1.0.lcm
Date Acquired : 2023/8/2 15:27:57

Chromatogram

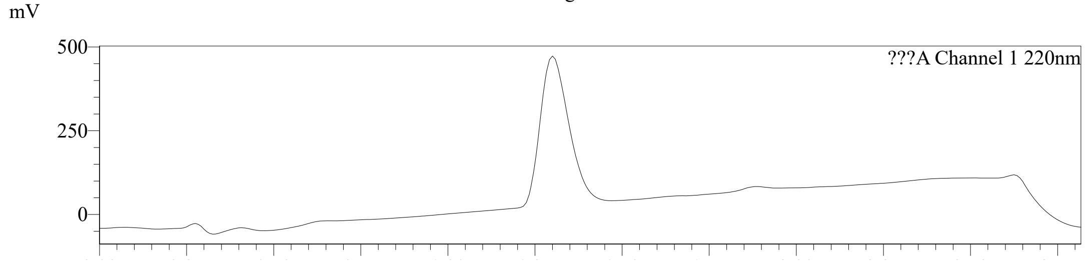

Peak Table

???A Channel 1 220nm

Peak#	Ret. Time	Area	Height	Conc.	Unit	Mark	Name
Total							

???A Channel 2 254nm

Peak#	Ret. Time	Area	Height	Conc.	Unit	Mark	Name
Total							

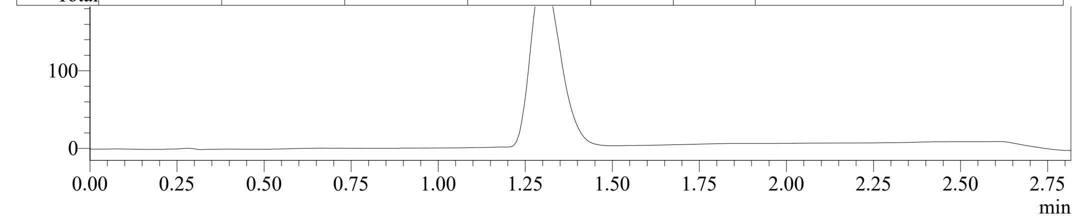

MS Spectrum Graph

Line#:1 R.Time:1.300(Scan#:79)
MassPeaks:961
Spectrum Mode:Single 1.300(79) BasePeak:570.10(13870946)
BG Mode:None Segment 1 - Event 1
ESI Positive

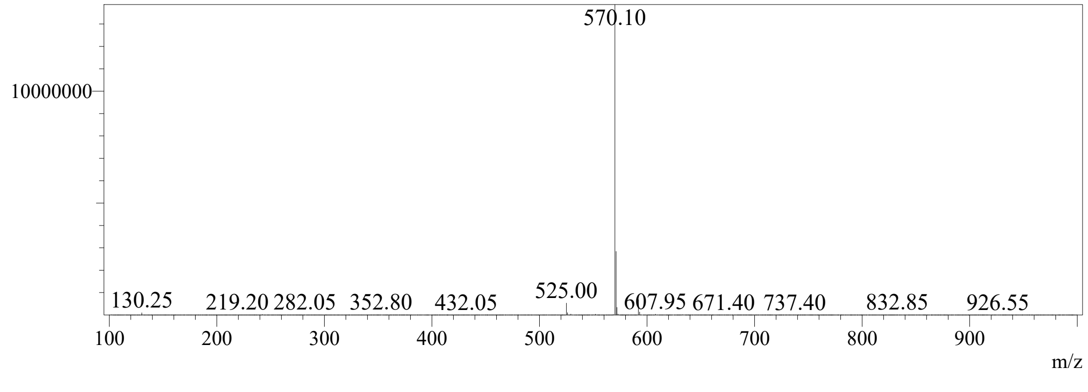

Line#:2 R.Time:1.316(Scan#:80)
MassPeaks:946
Spectrum Mode:Single 1.316(80) BasePeak:613.90(413217)
BG Mode:None Segment 1 - Event 2
ESI Negative

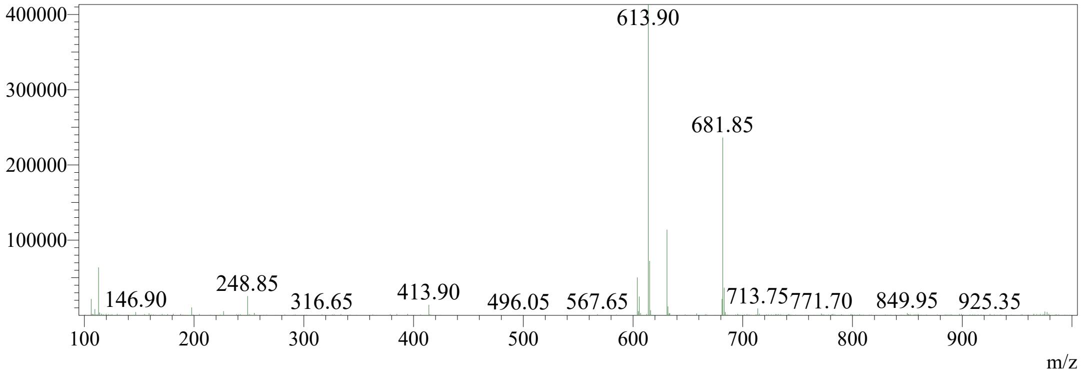

LC-MS, NMR and chiral column data for RTx-303

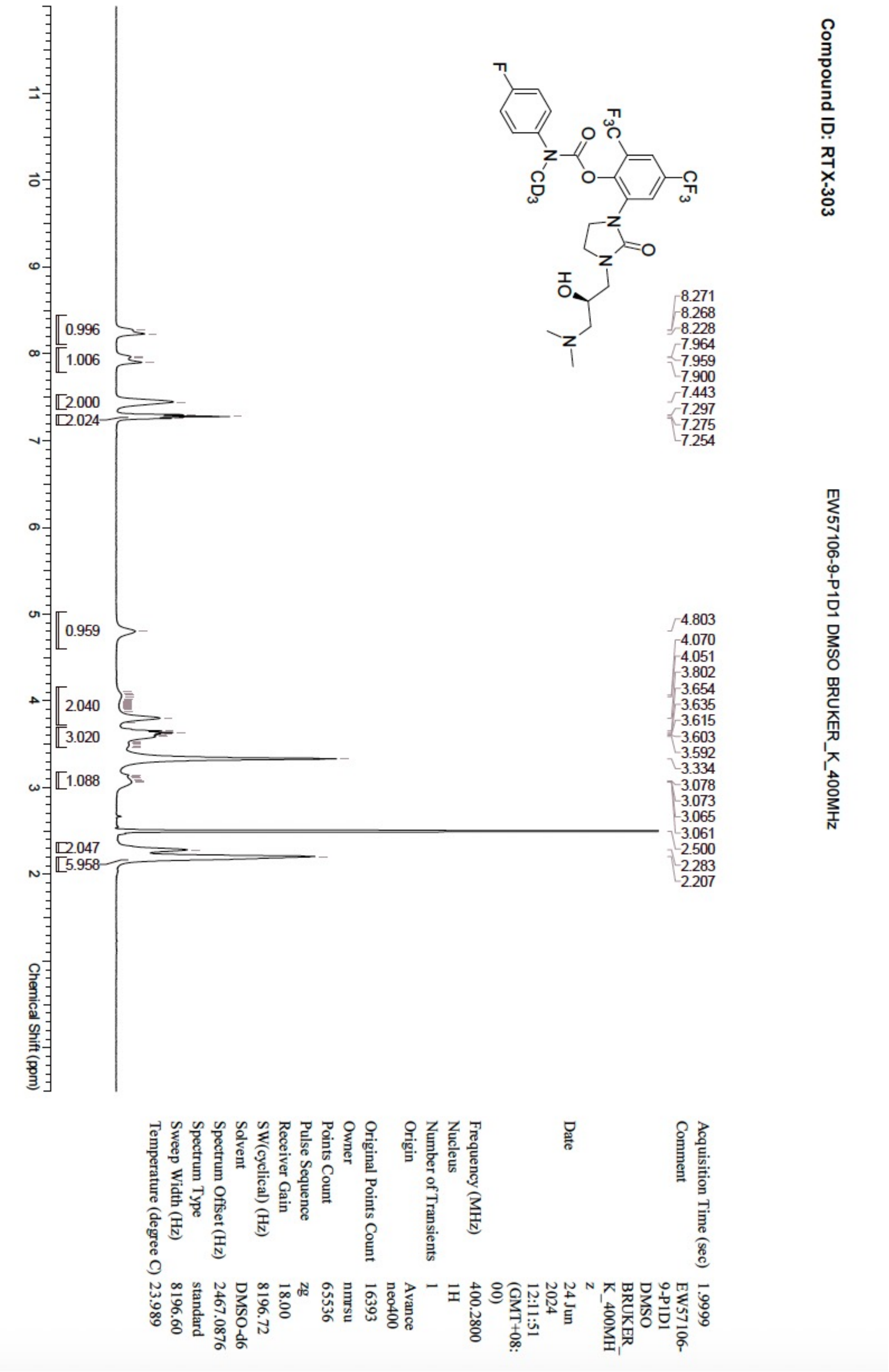

LCMS REPORT

Compound ID : 1
Sample ID : EW57106-9-P1D1
Injection Vol : 4ul
Location : vial24
Tray Name :2
Acq Method : D:\method\5-95AB_3min_220&254.lcm
Org DataFile : D:\DATA\2024\2406\240624\EW57106-9-P1D1.lcd
Injection Date : 6/24/2024 12:26:35
Instrument : LCMS-059 7-129

Chromatogram

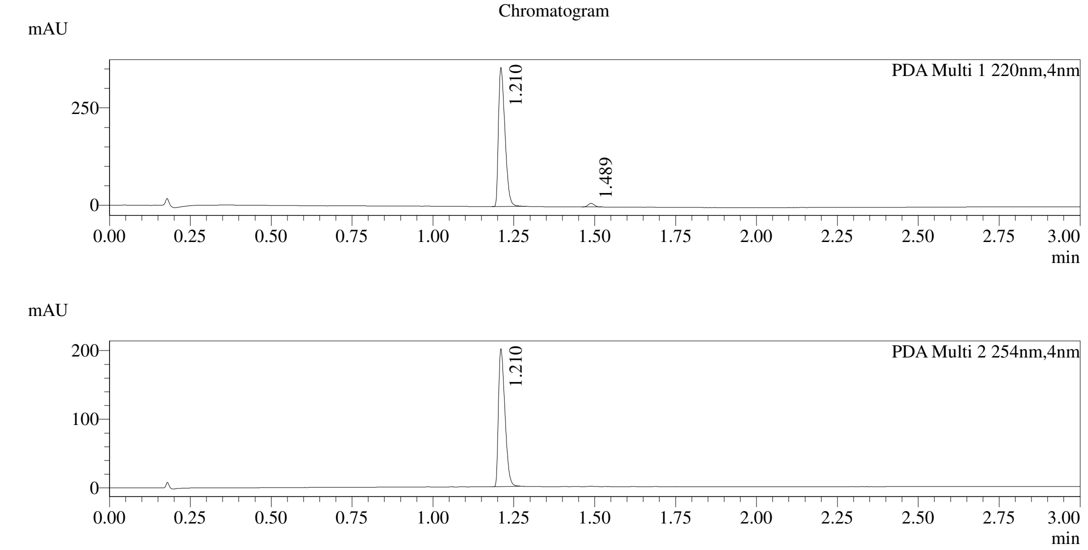

1 PDA Multi 1 / 220nm,4nm
2 PDA Multi 2 / 254nm,4nm

MS Chromatogram

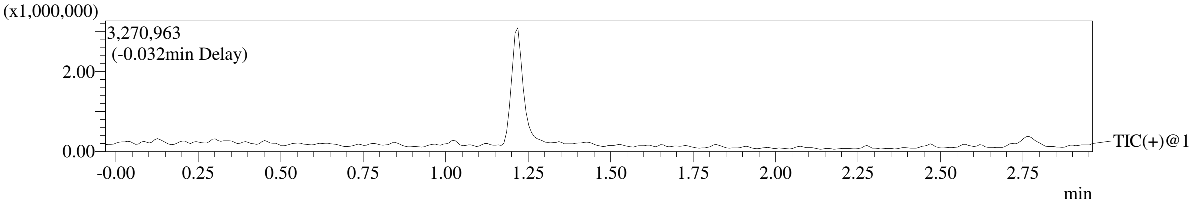

Integration Result

Peak Table

PDA Ch1 220nm

Peak#	Ret. Time	Height	Height%	USP Width	Area	Area%
1	1.210	356874	97.461	0.039	497806	97.569
2	1.489	9298	2.539	0.037	12401	2.431

Peak Table

PDA Ch2 254nm

Peak#	Ret. Time	Height	Height%	USP Width	Area	Area%
1	1.210	200730	100.000	0.039	279723	100.000

Mass Spectrum

RetTime: 1.210   Datafile: D:\DATA\2024\2406\240624\EW57106-9-P1D1.lcd

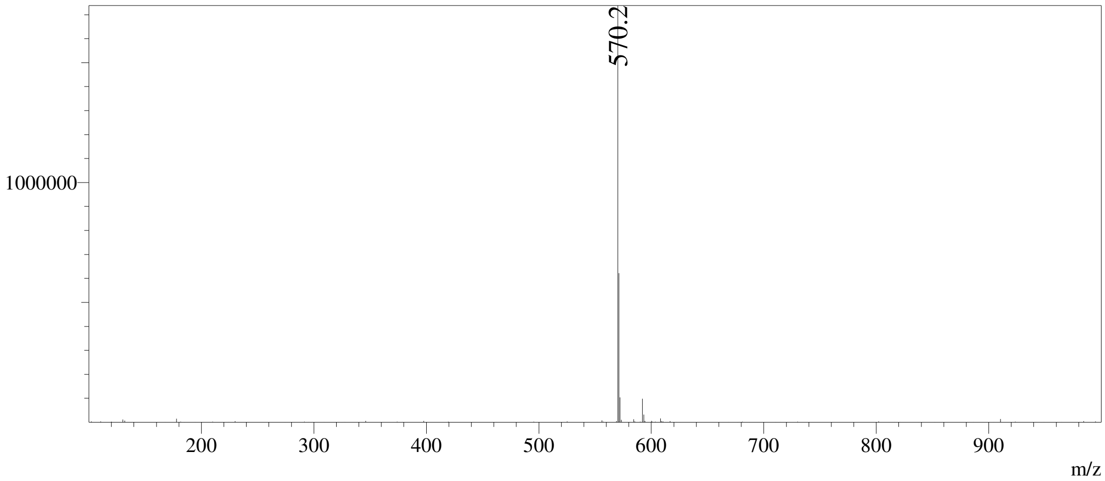

RetTime: 1.485   Datafile: D:\DATA\2024\2406\240624\EW57106-9-P1D1.lcd

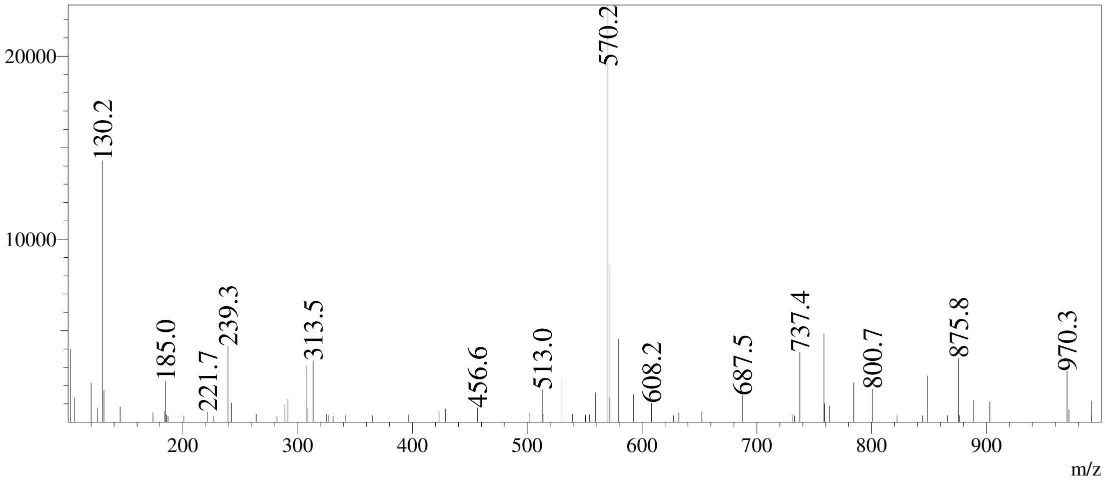

Chiral SFC Report

Compound ID : RTX-303
Sample ID : EW57106-9-P1A_J1
Injection Vol : 5ul
Location :Tray2 vial19
Acq Method : D:\METHODS\IG-3-EtOH(DEA)-5-7MIN-3ML-35T.lcm
Raw Data : D:\DATA\2024\202406\20240624\EW57106-9-P1A_J1.lcd
Injection Date : 6/24/2024 4:34:20 PM
Instrument : CAS-WH-ANA-SFC-J(SHIMADZU LC-30ADsf)

Method details: "Column:Chiralpak IG-3 50*4.6mm I.D.,3um
Mobile phase:Phase A for CO2,and Phase B for EtOH(0.05%DEA);
Gradient elution: 5% EtOH(0.05%DEA) in CO2 ,
Flow rate:3mL/min;Detector:PDA;
Column Temp:35C;Back Pressure:100Bar"

Chromatogram

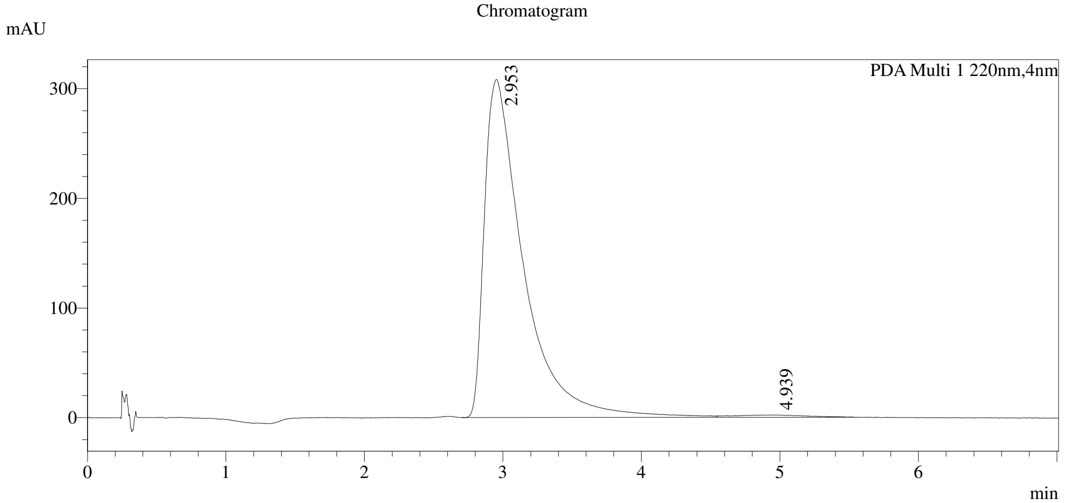

1 PDA Multi 1 / 220nm,4nm

==
Integration Result
==

Peak Table

PDA Ch1 220nm

Peak#	Ret. Time	Height	Height%	Resolution(USP)	Area	Area%
1	2.953	308086	99.306	--	6039389	98.739
2	4.939	2152	0.694	3.159	77122	1.261
